# Evolution of four gene families with patchy phylogenetic distributions: influx of genes into protist genomes

**DOI:** 10.1186/1471-2148-6-27

**Published:** 2006-03-21

**Authors:** Jan O Andersson, Robert P Hirt, Peter G Foster, Andrew J Roger

**Affiliations:** 1 Institute of Cell and Molecular Biology, Uppsala University, Biomedical Center, Box 596, S-751 24 Uppsala, Sweden; 2 School of Biology, The Devonshire Building, The University of Newcastle upon Tyne, NE1 7RU, UK; 3 Department of Zoology, The Natural History Museum, Cromwell Road, London SW7 5BD, UK; 4 The Canadian Institute for Advanced Research, Program in Evolutionary Biology, Department of Biochemistry and Molecular Biology, Dalhousie University, Halifax, Nova Scotia B3H 1X5, Canada

## Abstract

**Background:**

Lateral gene transfer (LGT) in eukaryotes from non-organellar sources is a controversial subject in need of further study. Here we present gene distribution and phylogenetic analyses of the genes encoding the hybrid-cluster protein, A-type flavoprotein, glucosamine-6-phosphate isomerase, and alcohol dehydrogenase E. These four genes have a limited distribution among sequenced prokaryotic and eukaryotic genomes and were previously implicated in gene transfer events affecting eukaryotes. If our previous contention that these genes were introduced by LGT independently into the diplomonad and *Entamoeba *lineages were true, we expect that the number of putative transfers and the phylogenetic signal supporting LGT should be stable or increase, rather than decrease, when novel eukaryotic and prokaryotic homologs are added to the analyses.

**Results:**

The addition of homologs from phagotrophic protists, including several *Entamoeba *species, the pelobiont *Mastigamoeba balamuthi*, and the parabasalid *Trichomonas vaginalis*, and a large quantity of sequences from genome projects resulted in an apparent increase in the number of putative transfer events affecting all three domains of life. Some of the eukaryotic transfers affect a wide range of protists, such as three divergent lineages of Amoebozoa, represented by *Entamoeba*, *Mastigamoeba*, and *Dictyostelium*, while other transfers only affect a limited diversity, for example only the *Entamoeba *lineage. These observations are consistent with a model where these genes have been introduced into protist genomes independently from various sources over a long evolutionary time.

**Conclusion:**

Phylogenetic analyses of the updated datasets using more sophisticated phylogenetic methods, in combination with the gene distribution analyses, strengthened, rather than weakened, the support for LGT as an important mechanism affecting the evolution of these gene families. Thus, gene transfer seems to be an on-going evolutionary mechanism by which genes are spread between unrelated lineages of all three domains of life, further indicating the importance of LGT from non-organellar sources into eukaryotic genomes.

## Background

During the past five years a number of reports have appeared indicating that protists acquire genes via LGT [1-4]. Recently, phylogenomic analyses of the complete genome sequences of *Entamoeba histolytica *and *Cryptosporidium parvum *indicated that several genes of these human parasites, including some key metabolic enzymes, most likely had been acquired from prokaryotes. 96 cases of relatively recent LGT from prokaryotic sources were reported for the former and 24 for the latter [5,6]. There are reasons to believe that LGT actually does influence protist genome evolution, since foreign genetic material is constantly entering the cell via food organisms. In addition, many protists harbour prokaryotes or eukaryotes (such as those that gave rise to secondary and tertiary plastids [7]) as endosymbionts. As a result, the occasional incorporation of genes from engulfed cells into the nucleus may facilitate a process of directional transfer of genes from the food organisms to phagotrophic eukaryotes over evolutionary time [4,8]. There is a growing amount of data that are consistent with this hypothesis. For instance, LGT has mostly been detected in phagotrophic lineages [4,9]. Moreover, the introduced genes in these lineages seem to have originated from organisms sharing the same environment with the recipient organisms – the anaerobic diplomonad lineage was found to have acquired genes from anaerobic prokaryotes in most cases [10], 22% of candidate donors lineages in LGT cases for *Entamoeba histolytica *involve relatives of the Bacteroides group which are abundant in human digestive tract [5], while the alga *Bigelowiella natans *has acquired genes mostly from other algae [9]. These observations are consistent with the idea that physical proximity in the environment of the donor and recipient lineages may greatly enhance the probability of a successful gene transfer event [11], a notion recently supported by phylogenetic analyses of 144 prokaryotic proteomes identifying gene pools shared between organisms (including distantly related one) occupying the same ecological niche [12].

Most of the claims of LGT in protists are based on unexpected phylogenetic relationships between protist and prokaryotic sequences [2,4]. However, phylogenetic methods are susceptible to systematic error that could lead to false interpretations of transfer events [2]. For example, a recent phylogenetic analysis indicated that the hydrogenosomal NuoF protein from *Trichomonas vaginalis *(a subunit of respiratory chain complex I) branched outside of a clade of mitochondrial homologs [8], leading the authors to propose a separate (non-mitochondrial) origin for this protein. However, these analyses failed to take into account the heterogeneity of amino acid (aa) composition displayed by sequences in this dataset [13]. In contrast, when the dataset is analysed with methods designed to avoid this potential artefact, the *T. vaginalis *sequence branched within the mitochondrial cluster [14], in agreement with the well-supported hypothesis that *Trichomonas *hydrogenosomes share an evolutionary origin with mitochondria [15,16]. Similarly, Cpn60 phylogenies with different taxonomic samplings led to important differences in the phylogenetic relationships amongst anaerobic protists including *E. histolytica *and two diplomonads (*Giardia *and *Spironucleus*) eliminating the possibility of an LGT event between *Entamoeba *and *Giardia *lineages[2,17]. In both these cases, extreme divergence coupled with compositional biases in these sequences suggested, correctly, their unexpected branching patterns were due to phylogenetic artefacts. In contrast, the phylogenetic analyses of the alanyl and prolyl tRNA synthetases show the expected phylogenetic relationships amongst prokaryotes and eukaryotes with the exception that several protist sequences were found nested within Archaea as sisters to the Nanoarchaeota sequences. In this case, the observations could not be attributed to any known phylogenetic artefacts and were most easily explained in [18] as gene transfer events from the archaeal lineage to the protists.

Interpretations of phylogenetic analyses of proteins with a more patchy distribution in the tree of life are more challenging than the cases described above. For example, gene duplications followed by differential gene loss may also yield the unexpected phylogenetic relationships that are hallmarks of LGT. In addition, genes with a patchy distribution may only be present in one or a few lineages in each organismal group making it potentially more difficult to identify donor and recipient lineages of gene transfer events since such assignments require that recipient lineages are nested within the donor group. Fortunately, the number of complete genome sequences is steadily growing, and should clarify the patterns of gene distribution within the tree of life. In combination with thorough phylogenetic studies, analyses of the presence and absence of genes in completely sequenced genomes should be very able to differentiate putative cases of gene transfers in gene families with a patchy phylogenetic distribution from other scenarios [19].

To investigate whether phylogenetic artefacts, and/or unappreciated gene duplication and loss events, have influenced previous interpretations of LGT, we have broadened the taxon sampling of four gene families with patchy phylogenetic distributions, previously implicated in gene transfer events in diplomonads and *E. histolytica *[10]. The updated datasets – *priS *(encoding a hybrid-cluster protein), *fprA *(A-type flavoprotein), *nagB *(glucosamine-6-phosphate isomerase), and *adhE *(alcohol dehydrogenase E) – were also analysed using more sophisticated phylogenetic methods. We have previously argued that these four genes were introduced into the genomes of diplomonads and *Entamoeba *from different sources based on phylogenetic analyses [10]. If these previous observations were really indicative of LGT, increased sampling of eukaryotic and prokaryotic taxa should result in an equal or increased number of distinct eukaryotic groups in the phylogenetic analyses (i.e. eukaryotes would be polyphyletic) and stronger support for tree topologies consistent with LGT. Alternatively, a different pattern is expected if the interpretation of gene transfers were based on phylogenetic artefacts and/or differential losses. In the former case, increased taxonomic sampling should, if anything, provide evidence for a common ancestry for the diplomonad and *Entamoeba *sequences – as improved within-clade taxonomic sampling tends to improve phylogenetic accuracy [20] – reducing the number of independent eukaryote groups observed. Alternatively, if the 'polyphyletic eukaryotes' pattern was due to ancient duplications and poor paralog sampling, we would expect newly sampled sequences to cluster in the different eukaryotic clades and recover mirror eukaryotic phylogenies.

To test these alternative hypotheses, we focused our active sampling of taxa to relatives of *Entamoeba*; the amphizoic *E. moshkovskii*, the turtle parasite *E. terrapinae*, the snake parasite *E. invadens*[21,22], and the more distantly related free-living amoeboflagellate *Mastigamoeba balamuthi *[23,24], and a putative relative of diplomonads; the parabasalid *Trichomonas vaginalis *[18,25-27] (the cause of trichomoniasis, a sexually transmitted disease in humans [28]). In addition, we updated our datasets with all currently available homologous sequences in the public databases as well as from a number of ongoing genome sequencing projects of eukaryotes. Our updated phylogenies using more sophisticated models of aa substitutions in combination with analyses of the distribution pattern of the genes indicate that gene transfer hypotheses currently best explain the data.

## Results and discussion

### Patchy distribution in eukaryotes

**Figure 1:**
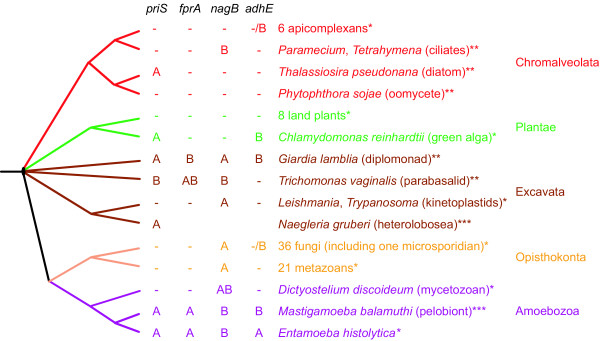
**Distribution of the four genes in the taxa sampled in this study. **A hypothetical tree of eukaryotes for which genomes have been fully sampled and published, *; is close to completion, **; or only partially sampled (genome sequence survey or expressed sequence tags), ***; indicating their classification into "super-groups" [29-31], showing the presence or absence of the four genes in the study. Please notice that the gene absences in the genomes that are close to completion are unconfirmed, they may turn into presences upon publication. A and B refer to strongly separated groups in the phylogenetic analyses, as indicated in Figures 2-4 & 6. The *priS *genes encode the hybrid-cluster proteins, *fprA *genes encode the A-type flavoproteins, *nagB *genes encode glucosamine-6-phosphate isomerase proteins and the *adhE *genes encode the alcohol dehydrogenase E proteins.

In a previous study we identified diplomonad genes potentially derived from LGT [10]. Here we have tested if alternative hypotheses and/or phylogenetic artefacts could account for these observations. We have broadened the eukaryotic taxon sampling of four genes with a limited distribution among eukaryotes, both by cloning and sequencing new genes and mining of the available sequence databases. By using this approach we are also able to refine the timing of putative LGT events with respect to organismal divergences and to gain insights into the evolution of gene families with a patchy distribution in general. All four genes were obtained from *Entamoeba invadens*, *Entamoeba moshkovskii *and *Entamoeba terrapinae*, and *priS*, *fprA *and *nagB *(partial) sequences were obtained from *Mastigamoeba balamuthi *with PCR using genomic DNA as template. Two *T. vaginalis *cDNA clones (*nagB *and *fprA*) were also completely sequenced. A *T. vaginalis priS *sequence and a *M. balamuthi adhE *sequence had appeared in the databases since the previous analysis. Furthermore, a *N. gruberi priS *cDNA clone was completely sequenced (see Additional File 1 for complete listing of the datasets). To further investigate the distribution of these genes in complete or nearly complete genome sequences, we performed similarity searches against available data from ongoing eukaryotic genome projects and retrieved the significant BlastP hits. We also combined these results with the information from published genomes and mapped the occurrences of the genes onto the current hypothesis of organismal relationships among eukaryotes (Figure 1) [29-31]. The four genes show a very patchy distribution often with both presences and absences within the same eukaryotic "super group". Two extreme alternative explanations may be invoked to explain these distribution pattern within eukaryotes; (i) presence of all four genes in the last common eukaryotic ancestor followed by many differential losses within the "super groups", or (ii) absence of the genes in the ancestor followed by independent gene acquisitions in all divergent lineages that possess the genes. The duplication and gene loss scenario becomes less likely the more independent convergent gene loss events need to be postulated. Therefore, phylogenetic analyses of the individual genes should help to distinguish between these hypotheses.

### Phylogenetic analyses

**Figure 2:**
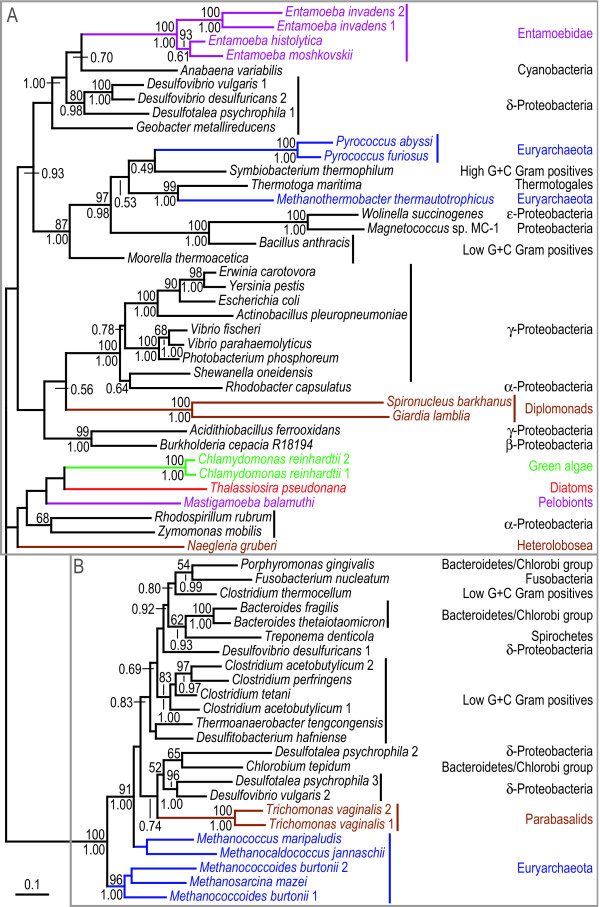
**Protein maximum likelihood tree of hybrid-cluster protein (*priS *gene). **ML tree based on 417 unambiguously aligned aa positions of the hybrid-cluster protein. Bootstrap support values >50% from ML analyses are shown above the branches. Posterior probabilities for the Bayesian consensus tree of the grouped aa analysis are shown below the branches. When no space is available a line indicates the position of the support values. Absence of a posterior probability value at a node indicates that this node was lacking in the Bayesian consensus tree. Details about the phylogenetic analyses are found in the Methods section and AdditionalAdditional File 2. The grey boxes A and B indicate strongly separated groups which include eukaryotic sequences. The tree is arbitrarily rooted. Eubacteria are labelled black, Archaea are labelled blue, and the Eukaryotes are labelled according to their classification into "super-groups" [29, 30]: opisthokonts (orange), amoebozoa (purple), chromalveolates (red), plants (green) and excavates (brown) (see Figure 1).

**Figure 3:**
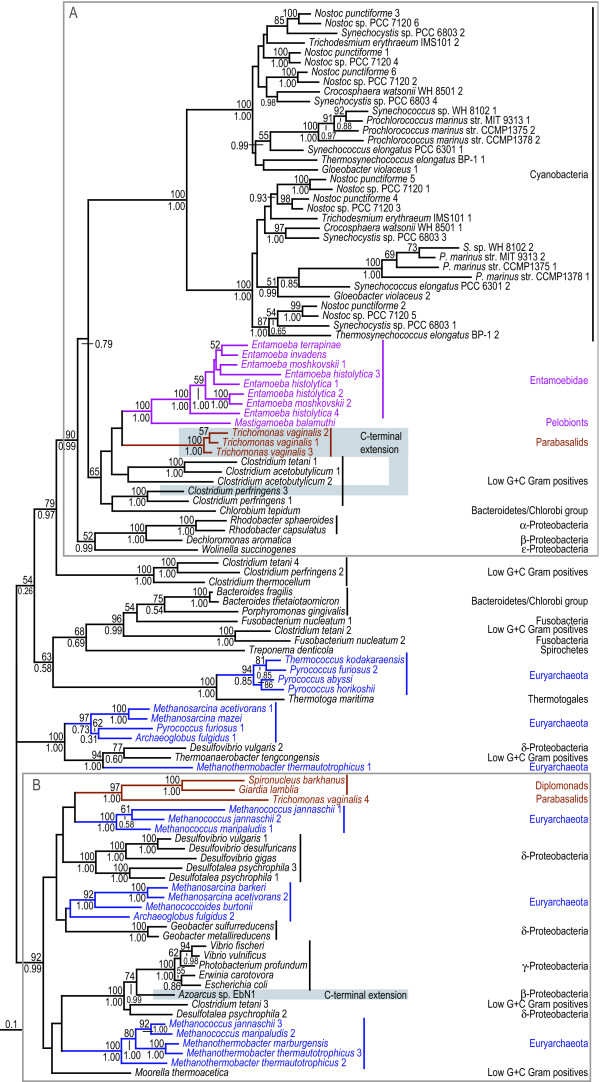
**Protein maximum likelihood trees of A-type flavoprotein (*fprA *gene). **ML trees based 269 unambiguously aligned aa positions of the A-type flavoprotein. The boxes indicate sequences that have an approximately 450 aa long conserved C-terminal extension of the flavoprotein which is absent from all other sequences in the alignment (see Additional File 4 for further analyses and discussion). The grey boxes A and B indicate strongly separated groups which include eukaryotic sequences. The tree is arbitrarily rooted. Details about the phylogenetic analyses are found in the Methods section and Additional File 2. Labelling as in Figure 2.

**Figure 4:**
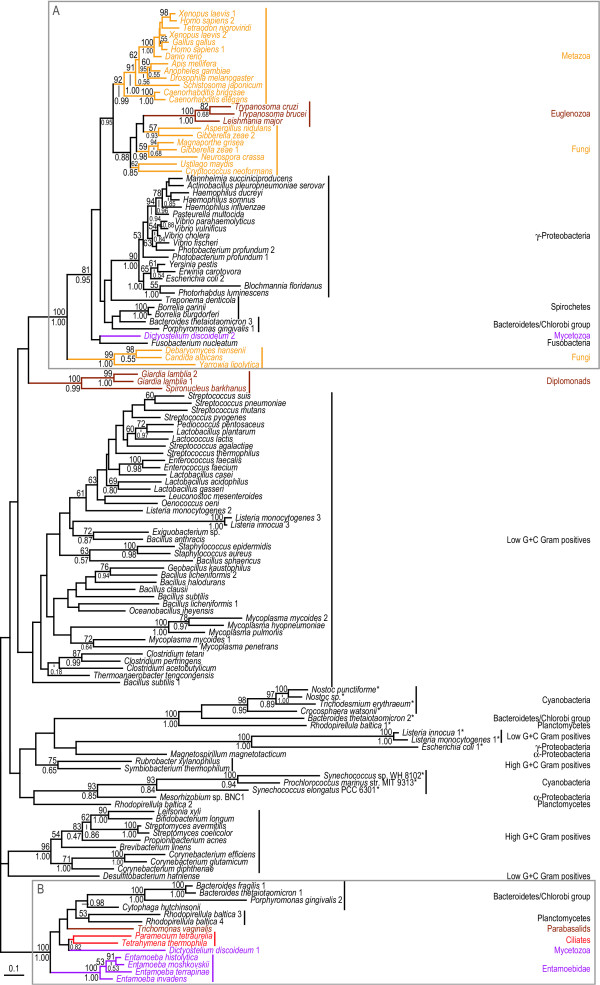
**Protein maximum likelihood trees of the short and long versions of glucosamine-6-phosphate isomerase (*nagB *gene)**. ML tree based on 229 unambiguously aligned aa positions from the N-terminal part of the alignment of the glucosamine-6-phosphate isomerase protein. The grey boxes A and B indicate strongly separated groups which include eukaryotic sequences. The sequences in the B box (with the exception of the *R. baltica *3 sequence) have an approximately 500 aa long conserved C-terminal extension of the protein which is absent from all other sequences in the alignment. The sequences in box B, together with the sequences indicated with asterisks were excluded in a separate analysis shown in Additional File 5, to test the influence of the removal of the long version of the protein and long branches on the relative positions of eukaryotic sequences. The tree is arbitrarily rooted. Details about the phylogenetic analyses are found in the Methods section and Additional File 2. Labelling as in Figure 2.

**Figure 5:**
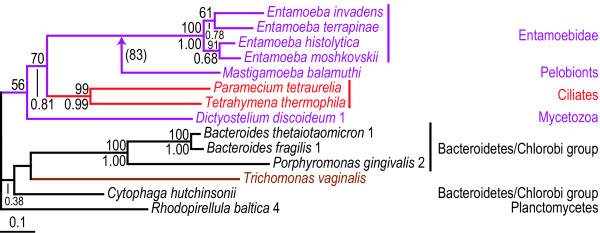
**Protein maximum likelihood trees of the long version of glucosamine-6-phosphate isomerase (*nagB *gene)**. Phylogenetic tree based on 560 unambiguously aligned aa positions from the glucosamine-6-phosphate isomerase sequences that have the long C-terminal extension (box B in Figure 4). In a separate analysis the partial *Mastigamoeba balamuthi *sequence was included and its position is indicated with an arrow with the bootstrap support value in parenthesis. The tree is arbitrarily rooted. Details about the phylogenetic analyses are found in the Methods section and Additional File 2. Labelling as in Figure 2.

**Figure 6:**
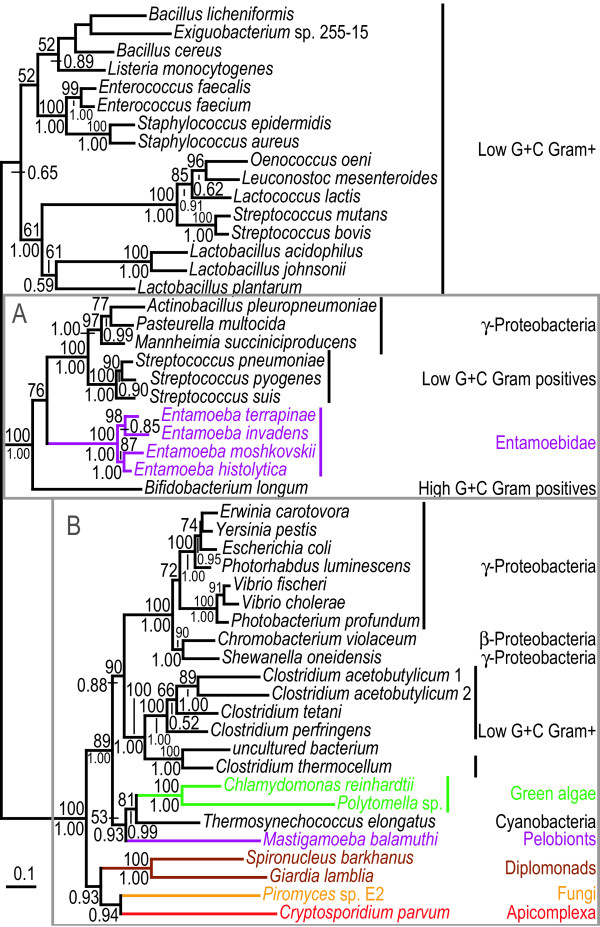
**Protein maximum likelihood tree of alcohol dehydrogenase E (*adhE *gene). **Phylogenetic tree based on 796 unambiguously aligned aa positions of the alcohol dehydrogenase E protein sequences. The grey boxes A and B indicate strongly separated groups which include eukaryotic sequences. The tree is arbitrarily rooted. Details about the phylogenetic analyses are found in the Methods section and Additional File 2. Labelling as in Figure 2.

In our previous study we excluded sequences that showed indications of a biased aa composition to reduce the impact of phylogenetic artefacts due to compositional heterogeneity where possible – the available methods at the time assumed aa compositional homogeneity [10]. Here we approach this potential problem by including analyses with methods and models that are designed to mitigate the potential misleading effects of compositional heterogeneity. Each aa in the alignments was recoded to the six groups of chemically related aa that commonly replace one another [32,33], an approach identical to the recent analyses of the NuoF protein [14]. Previously, we were also limited by the size of the datasets [10], since the maximum likelihood (ML) methods were very computationally demanding at the time. The release of the PHYML software solves this problem since it is able to perform bootstrap analyses of a large number of sequences (>100) in a reasonable computational time [34]. The recently released ModelGenerator software also ensures the usage of the optimal available model for aa substitutions in the ML analyses [35]. These advances in the field of phylogenetics enabled us to perform more detailed analyses that include all available members of each gene family. Information about the datasets and parameters for the phylogenetic analyses are listed in Additional File 2, and the phylogenetic trees with support values from the two methods are shown in Figures 2, 3, 4, 5, 6.

All datasets in the phylogenetic analyses with grouped aa using the Metropolis-coupled Markov Chain Monte Carlo (MCMC) strategy showed convergence, indicated by good agreements between the split support values of the duplicate runs (Additional File 3). Two of the alignments (the long version of glucosamine-6-phosphate isomerase and the prismane protein) also showed a good model composition fit indicated by both posterior predictive simulations and tests for homogeneity using *X*^2 ^statistics and simulations to get the null distribution (*p*_*t *_> 0.05 and P_sim _> 0.05, respectively) [36], while the original datasets did not (P_sim _< 0.05) (Additional File 2). This indicates that the recoding procedure has reduced the potential misleading effects of compositional heterogeneity in these two analyses. The other three datasets (A-type flavoprotein, the short version of glucosamine-6-phosphate isomerase, and alcohol dehydrogenase E) showed low *p*_*t *_and P_sim _values (<0.05), suggesting that compositional heterogeneity might still represent a source of artefactual results in these datasets (Additional File 2). Nevertheless, none of these grouped aa datasets failed the tests of the model composition when the χ^2 ^curve was used to get the null distribution, while two of the these three original datasets did, suggesting that the recoding procedure had improved the model fit (Additional File 2), reducing the potential for estimation biases. At the very least, these analyses complement the more "standard" ML analyses by showing what aspects of the phylogenies are robust to aa recoding and reducing any potential effects of saturation.

The updated phylogenetic analyses show sequences highly scrambled with respect to expected organismal relationships (Figures 2, 3, 4, 5, 6), as previously observed for these genes [10]. Thus, the earlier finding that these proteins produce phylogenetic trees that are incompatible with organismal phylogenies is robust with respect to improved taxon sampling and more detailed phylogenetic analyses – the number of eukaryotic groups (polyphyly) in the trees have increased, rather than decreased. In all analyses the eukaryotic sequences are found in at least two distinct regions of the trees nested with prokaryotic sequences (A and B boxes in Figures 2, 3, 4 &6), which are separated with strong support values. These strong separations could, in principle, be due to ancient duplication events followed by a large number of differential losses. Indeed, the presence of the same prokaryotic group in both regions in several of the phylogenetic analyses – low G+C Gram positives are for example found in both box A and B in Figures 2 and 6 – superficially supports ancient duplications. Such scenarios are expected to result in phylogenetic relationships for each paralog that mirror the organismal relationships. This is not observed in our analyses (Figures 2, 3, 4 &6). Furthermore, duplication and loss scenarios require that the gene was present in multiple copies in the last common universal ancestor and retained for a long evolutionary time. Thus, to explain the patterns we observe in the phylogenies (Figures 2, 3, 4, 5, 6) a eukaryotic ancestral genome that encoded a larger number of distantly related paralogs of the four genes than present in any of the extant eukaryote genomes would have to be inferred (Figure 1). To our knowledge, no data exist supporting a universal trend for drastic genome shrinkage in a relative recent evolutionary time. Therefore, gene duplication and differential losses alone do not seem sufficient to explain the unexpected phylogenetic relationships observed in our analyses (Figures 2, 3, 4, 5, 6).

However, the number of independent gene losses has to be weighed against the possibility of a later introduction of the genes into eukaryotes by LGT events. Yet, as none of the eukaryotic groups are found nested within a natural prokaryotic group with strong bootstrap support (Figures 2, 3, 4, 5, 6), it is difficult to identify donor and recipient lineages involved in the putative LGT events. Thus, the presence of these genes in a subset of the sampled eukaryotes are neither easily explained by vertical inheritance of the genes from the common ancestor of all eukaryotes, nor by a distinct number of easily identified gene transfer events. These phylogenies need to be carefully interpreted in combination with analysis of gene distribution patterns, as well as in the context of the biology of the available organisms.

### Hybrid-cluster protein

Genes for the hybrid-cluster protein (*priS*) have been identified from a large number of prokaryotes, as well as several eukaryotes (Figures 1 &2). However, the cellular function of the protein is not well established; potential roles in the biological nitrogen cycle [37,38] and the adaptive response to oxidative stress [39] have been suggested. Although the gene is found in all three domains of life, its distribution within the domains are patchy; for example, it is relatively widespread among proteobacterial genomes, while it has only been found in a single high G+C Gram positive species and a single cyanobacterium (Figure 2). The occurrence of the gene in a large number of unrelated lineages in combination with the absence from more closely related species is most simply explained by cross-species transmission via gene transfer. Indeed, the phylogeny of the hybrid-cluster protein strongly suggests a number of intra- and inter-domain prokaryotic LGT events, with sequences from organismal groups such as proteobacteria, low G+C Gram positives, and euryarchaeota branching in several distinct regions of the tree, often branching with unrelated lineages with strong support values (Figure 2). The eukaryotes are found within two large groups of sequences including both archaeal and bacterial homologs, separated by a long and strongly supported branch (box A and B in Figure 2). One clade contains two *Trichomonas *sequences that are the sole eukaryotes in one of these groups (box B in Figure 2). A prokaryote-to-eukaryote LGT event affecting the parabasalid lineage after the divergence from other eukaryotes, including diplomonads, is a more parsimonious explanation for the position of the *T. vaginalis *sequences than loss of this version of the gene in all other eukaryotic species, provided *T. vaginalis *is not basal to all the other eukaryotes included in this analysis [18,25-27]. However, the prokaryotic donor lineage for the *T. vaginalis *sequences is difficult to determine from the current data and analyses.

The eukaryotic sequences found in the second group within the hybrid-cluster protein phylogeny are found in four polyphyletic groups (box A in Figure 2). However, only one of these groups is separated from the other with a significant statistical support; the *Entamoeba *sequences form a weakly supported group with a cyanobacterial sequence which are found as a sister group of three δ-proteobacterial sequences with a posterior probability of 1.00 in the grouped aa analysis. This is suggestive of a eubacteria-to-*Entamoeba *LGT event, perhaps with cyanobacteria or δ-proteobacteria as the donor lineage (Figure 2). Thus, at the very least the phylogeny of the hybrid-cluster protein suggests two transfer events from prokaryotic donors into protists. Taken at face value, the tree also supports additional transfers into various protist lineages. Indeed, the diplomonad lineages are nested within proteobacterial sequences in both the ML and grouped aa Bayesian analyses, although with weak statistical support in both cases. At any rate, this observation is suggestive of a LGT event from a proteobacterium to the diplomonad lineage. Two α-proteobacterial sequences are nested within the other eukaryotic *priS *sequences in box A with weak bootstrap support (Figure 2), which could indicate an origin via endosymbiotic gene transfer. However, the absence of the gene in mitochondrial genomes in combination with its absence from the nuclear genome of most eukaryotes related to pelobionts, diatoms, heterolobosea, and green algae (Figure 1), makes such an origin doubtful. Still, the weakly supported separation of diplomonad and these eukaryotic sequences may be artefactual – in reality they could represent a monophyletic group that inherited this gene from their common ancestor. If so, at least eight independent losses of *priS *in the apicomplexan/ciliate, oomycete, land plant, parabasalid, kinetoplastid, opisthokont, mycetozoan, and *Entamoeba *lineages would have to be invoked (Figure 1). Since such widespread and relatively recent independent losses appear unlikely, we favour a scenario where also the *N. gruberi*, *M. balamuthi*, *T. pseudonana*, and *C. reinhardtii *sequences have been distributed by an unknown number of gene transfer events from unsampled prokaryotic lineages or between microbial eukaryotic lineages. So far the *priS *gene has only been found in microbial eukaryotes. This circumstantially supports the hypothesis that the absence of a germ/soma separation in unicellular organisms increases their chance of acquiring genes by LGT [4]. We predict that additional taxon sampling will confirm the current trend of preferential presence in unicellular eukaryotes and will further clarify the origins of the eukaryotic *priS *genes.

### A-type flavoprotein

The *fprA *gene encodes A-type flavoprotein, a protein recently inferred to play a role in the detoxification of nitric oxide and/or oxygen in *E. histolytica *and was suggested to derive from a relatively recent LGT event from a prokaryotic donor [5]. Furthermore, it has been demonstrated that *T. vaginalis *is able to degrade nitric oxide under microaerophilic conditions, an activity proposed to be associated with the presence of A-type flavoproteins in these parasites [40]. Again, the gene is only found in a subset of the sequenced prokaryotes – mostly species able to grow in oxygen-poor environments (Figure 3). One exception is the widespread presence of the A-type flavoprotein within cyanobacteria, possibly indicating that the protein has evolved a different function within this group. Consistent with this hypothesis the cyanobacterial sequences are well separated from the other sequences in the tree and have a unique alignment feature; they all share a ~160 aa highly conserved C-terminal extension that is absent from all other sequences. The phylogenetic analyses of the A-type flavoprotein strongly indicate that the *fprA *gene has been distributed between the prokaryotic groups via LGT, rather than by vertical inheritance, since many groupings of unrelated prokaryotic taxa are observed and supported by strong support values from both analyses (Figure 3).

The eukaryotes are found in two clearly separated clusters. The two diplomonad sequences are found together with a *Trichomonas *sequence among a mixture of eubacterial and archaeal species, indicating a prokaryote-to-eukaryote gene transfer event to a hypothetical uniquely shared ancestor of diplomonads and parabasalids (box B in Figure 3) – unless several independent gene losses are inferred among a broad range of eukaryotic lineages (Figure 1). In the ML analysis, three additional *Trichomonas *homologs are found weakly associated with the strongly supported grouping of the *Mastigamoeba *and *Entamoeba *sequences (box A in Figure 3), while the *Entamoeba*/*Mastigamoeba *clade is found a sister clade to the *Clostridium perfringens *sequences in the grouped aa analysis with a posterior probability of 0.97 (data not shown). Thus, the relationships between amoebozoan, parabasalid and clostridial sequences are uncertain. However, *Trichomonas *does not share a recent common ancestor with amoebozoa (Figure 1) suggesting that the *fprA *gene has been acquired by separate gene transfer events in these two eukaryotic lineages. Alternatively, following a prokaryotic LGT to one of the two eukaryotic lineages, a second LGT took place between an ancestor of *Entamoeba *and a parabasalid. Interestingly, the three *T. vaginalis *sequences share a ~450 aa C-terminal extension of about 39% identity with the *Clostridium perfringens *3 *fprA *homolog (Figure 3 and Additional File 4). This sequence, and a 433 aa long *Clostridium tetani *sequence (an FAD-dependent pyridine nucleotide-disulphide oxidoreductase:Rubredoxin-type 38% identical to the *T. vaginalis *sequences) are the most similar prokaryotic sequences in the public databases, while the most similar eukaryotic sequence, an NADH dehydrogenase from *E. histolytica *previously identified to be of prokaryotic origin [41], is only 25% identical (Additional File 4). Such a taxonomic distribution of this protein domain links the *Trichomonas *C-terminal extensions with the *Clostridium *sequences rather than to the eukaryotic sequences, suggesting that they originated via a gene transfer event from a prokaryote donor. Thus, both the N-terminal and C-terminal domains of the *T. vaginalis *A-type flavoproteins likely have prokaryotic origins (see Additional File 4 for discussion of plausible scenarios).

The eukaryotic lineages that encode *fprA *are micro-aerophilic organisms that most likely have evolved from aerobic eukaryotes [15], and the prokaryotes found closest to the eukaryotic sequences in the tree are found in oxygen-poor environments. These observations indicate that the transfer of the gene occurred in such an environment. The putative functional role of *fprA *in nitric oxide detoxification [40] indicate that these gene transfers might represent metabolic adaptations that allowed these different eukaryotes to better survive in anoxic environments. *fprA *could be part of the gene pool shared between distantly-related organisms (prokaryotic or eukaryotic) that occupy the same ecological niche.

### Glucosamine-6-phosphate Isomerase

The *nagB *gene encodes glucosamine-6-phosphate isomerase, an enzyme which is usually about 260 aa residues in length and is required for the biosynthesis of the cyst wall in *Giardia *[42]. Apart from low G+C Gram positives and γ-proteobacteria, the *nagB *gene is only sparsely represented in eubacteria and not yet detected in archaea (Figure 4). It is also absent from several eukaryotic lineages (Figure 1). In the phylogenetic tree of *nagB*, a strongly supported group including the *Entamoeba*, ciliate, mycetozoa, pelobiont, parabasalid and several eubacterial sequences was detected (box B in Figure 4). All these sequences, with the exception of one of the *Rhodopirellula baltica *paralogs, have a roughly 500 aa residue long homologous C-terminal extension of the protein with pair-wise identities above 48%, which confirms the common ancestry of these sequences (Figure 4). To increase the resolution of this group, a separate analysis was performed which only included the sequences of the long version of the protein and therefore was based on a larger number of positions in the alignment – 560 unambiguously aligned aa residues compared to 229 (Figure 5). Interestingly, in both analyses, pelobiont and *Entamoeba *sequences form a group with the ciliate sequences. In the ML analyses the mycetozoan *Dictyostelium discoideum *is found as a sister to these sequences with a bootstrap support of 56% (Figure 5), while the *Rhodopirelulla baltica *4 sequence is found as the immediate outgroup to the ciliate/*Mastigamoeba*/*Entamoeba *sequences with a posterior probability of 0.45 in the grouped aa analysis (data not shown). These weakly supported and partly incongruent phylogenies could be rationalized in the following ways: (i) a phylogenetic artefact splitting the amoebozoa sequences, in combination with differential gene loss in all sampled eukaryotic genomes with only the currently sampled ciliates and amoebozoa retaining *nagB *(Figure 1), (ii) inter-domain gene transfers events from closely related, but yet unsampled, prokaryotes to the amoebozoa and ciliate lineages, or (iii) the presence of the long version of *nagB *in the common amoebozoan ancestor followed by a transfer event to the ciliate lineage. Although none of the alternatives can be excluded, we favour the third explanation, since the expected topology within the amoebozoa is recovered, albeit with only weak bootstrap support from the ML analysis (Figure 5), if a single intra-domain LGT event is inferred. Furthermore, ciliates are known to eat other protists [43,44], indicating that a gene transfer event from an amoebozoan to a ciliate is feasible at least in principle [8]. Further taxonomic sampling of eukaryotic and prokaryotic genomes is obviously needed, especially within the two particular eukaryotic groups concerned, to distinguish between the different plausible scenarios. In any case, the *Mastigamoeba *and *Entamoeba *sequences form a strongly supported group that indicates the presence of the gene in their common ancestor.

In contrast, the *Trichomonas *homolog, that encodes the long version of the enzyme, and diplomonads, that encode the short version, have distinct origins. While the parabasalid gene likely originated via a gene transfer event, possibly from a eubacteria within the Bacteroidetes/Chlorobi group (Figure 5), the source of the diplomonad genes remains uncertain. The separation from other eukaryotes in box A appear robust with strong bootstrap support from both of the analyses with all taxa (Figure 4), as well as an additional analysis where the box B sequences and prokaryotic long branches were excluded (Additional File 5). Thus, the separation of the diplomonad sequences from the eukaryotic sequences in box A is unlikely a result of long-branch attraction; an LGT event from an unsampled eubacterial lineage seems like a more likely explanation (Figure 4).

The topology of the tree relating the opisthokont sequences to other eukaryotic lineages and prokaryotes is not easy to explain simply by vertical inheritance (box A in Figure 4); the metazoan sequences are grouped together as expected, but the fungi are split into one main group and a smaller group with three budding yeast sequences. The separation between the two fungal groups is supported by both analyses (Figure 4). In fact, the budding yeasts are found with the other fungi only in 1% of the bootstrap replicates in the ML analyses (including the analysis without long branches) and never among the 2000 sampled trees in the grouped aa analysis. Furthermore, the eukaryotes within box A are never found as a monophyletic group among the 500 bootstrap replicates in any of the ML analyses, and with a posterior probability of only 0.02 in the grouped aa analysis (data not shown). Collectively, these results indicate that the fungal *nagB *genes likely have separate origins; a recent introduction of a *nagB *gene into a common ancestor of the three budding yeast lineages *Debaryomyces*, *Candida*, and *Yarrowia *seems like a reasonable scenario.

The *Dictyostelium *sequence is found as a sister to a *Fusobacterium *sequence in the two ML analyses (Figure 4 and Additional File 5), while the sequence is nested between the three budding yeast sequences and the other sequences in box A in Figure 4 in the grouped aa analysis with posterior probabilities for the separations of 0.95 and 0.90, respectively (data not shown). The separation to the metazoan/fungi/euglenozoan group is strong also in the ML analysis; the *Dictyostelium *sequence indeed never branches with this group in any of the bootstrap replicates in the full analysis or the analysis where long branches were excluded (data not shown). Accordingly, the phylogenetic analyses indicate that a gene acquisition from a prokaryotic lineage is a plausible explanation for the origin of the *Dictyostelium *sequence, rather than a shared ancestry with the other eukaryotic sequences within box A.

### Alcohol dehydrogenase E

Alcohol dehydrogenase E is a key enzyme in the energy metabolism of type I "amitochondriate" protists (i.e. those that lack energy-producing mitochondria or hydrogenosomes) [45], since it catalyzes the conversion of acetyl-CoA to ethanol in a two-step reaction which oxidizes two molecules of NADH to NAD^+ ^[46]. We expanded the dataset with *adhE *genes from three additional *Entamoeba*species. The failure to detect an *adhE *homolog in *T. vaginalis *in the ongoing genome project [47] was expected, since this organism contains hydrogenosomes (type II "amitochondrial" protist), and therefore utilizes a different set of enzymes in their energy metabolism [45]. Interestingly, alcohol dehydrogenase E genes have been detected in the anaerobic chytrid fungus *Piromyces *sp. E2, which indeed does contain hydrogenosomes [48]. However, energy metabolism of chytrids is clearly different from that of type II amitochondriate protists such as *Trichomonas *– chytrids exhibit a bacterial-type mixed-acid fermentation [48]. The finding of alcohol dehydrogenase E in two green algal species, where the protein functions in aerobic mitochondria, indicates that the diversity of its functional role in eukaryotes is not fully understood [49].

The phylogenetic tree supports our earlier interpretations that LGT has played an important role in the evolution of this gene [10,49], with a number of strongly supported prokaryotic relationships that most easily are explained by gene transfer events (Figure 6) – the gene is only rarely found outside low G+C Gram positives and γ-proteobacteria. The additional *Entamoeba *sequences form a group with the *E. histolytica *sequence, indicating the presence of the gene in the common ancestor of these *Entamoeba *species, while the *Mastigamoeba *sequence clearly has a distinct origin from that of its amoebozoan sisters, as observed previously [49]. The position of the *Entamoeba *sequences within a eubacterial group strongly suggests a prokaryote-to-eukaryote LGT event. Sequences from two diplomonads, two green algae, a single apicomplexan, and a chytrid fungus are found in the same region of the tree as the *Mastigamoeba *sequence (box B in Figure 6). However, relatives of these organisms are known to lack the gene (Figure 1), arguing in favour of recent independent introductions of the gene, rather than an ancestral presence followed by differential gene loss. The green algal sequences are found as sisters to the single cyanobacterial sequence (Figure 6) with moderate to strong statistical support from both analyses, indicating a transfer event between the lineages. This seems ecologically reasonable since ancestors of these lineages could have been found in the same environment. Although the observed topology could be explained by endosymbiotic gene transfer from the plastid, the fact that land plants are lacking the gene, the absence of the gene from extant plastid genomes, and the localization of the protein in green algal mitochondria [49] makes a gene transfer independent of the plastid endosymbiosis somewhat more likely. Also the *Mastigamoeba *sequence is separated from the other eukaryotic sequences in this region with strong and moderate support from the grouped aa and ML analyses, respectively (box B in Figure 6), clearly suggesting an origin via LGT, maybe from an unsampled prokaryotic lineage.

The diplomonad, fungal, and apicomplexan alcohol dehydrogenases are found in a weakly supported cluster in both analyses. This suggests eukaryote-to-eukaryote gene transfer events, although the donor and recipient lineages are difficult to infer. Indeed, it has earlier been suggested that the green algal *adhE *could have been acquired by the algae from parasitizing chytrid fungi or from foraminiferan hosts to endosymbiotic algae [49], and similar interactions between these lineages could be invoked to explain the exchange of *adhE *genes. Interestingly, *Cryptosporidium*, *Piromyces *and many diplomonads are all anaerobes or microaerophiles, and many share similar, if not identical, environments; the digestive tract of various mammals. This could have facilitated gene sharing via LGT between these distantly related eukaryotic lineages, although independent acquisitions from unsampled prokaryotes cannot be excluded. Interestingly, these three distantly related eukaryotic lineages most likely have adapted to an anaerobic lifestyle independently [15], and the putative acquisition of *adhE*likely represented independent metabolic adaptations to this environment. As for the *priS *gene (Figure 2), our prediction is that future genome sampling will only uncover *adhE *genes among microbial taxa since the distribution of *adhE *is restricted to microbial eukaryotes.

### LGT events as phylogenetic markers

LGT is usually expected to confound efforts to reconstruct organismal relationships, since it decouples the historical signals in the gene sequences from organismal lineages [50]. However, gene transfer events can also be informative in a specific case; the shared possession of a transferred gene may indicate a phylogenetic relationship between the lineages that possess the transferred gene to the exclusion of the lineages that lack it. There certainly are limitations for such interpretations; the gene could have been lost in some of the descendants of the recipient lineage and additional transfers can complicate the correct identification of donor and recipient lineages. In any case, gene transfer events are a potentially very important source of information about organismal relationships [18,51], especially for protists where the molecular data are scarce and phylogenetic reconstructions are difficult [52].

For example, the phylogenetic positions of pelobionts and *Entamoeba *have been difficult to resolve with molecular markers. Analyses of ribosomal RNA only weakly grouped these together [21], while more recently, based on a number of protein markers, it was conclusively shown that these two groups share a common ancestor to the exclusion of other eukaryotes [23,24]. Interestingly, the *Entamoeba *sequences strongly group together with the *Mastigamoeba *sequence in two of the four analyses discussed here, *fprA *and *nagB *(Figures 3 &5). This suggests that these genes were present in the ancestor of *Mastigamoeba *and *Entamoeba*, providing further support for a specific relationship between these two eukaryotic lineages. Furthermore, the higher eukaryotic taxon Amoebozoa [23,24] is reflected in the phylogeny of one of these genes, *nagB *(Figure 5), provided one accepts the recovered gene phylogeny that indicates a possible gene transfer from within this group to a ciliate lineage. If robust, this branching pattern could allow one to make inferences about the relative timing of divergences within Amoebozoa and the Ciliophora. However, improved taxonomic sampling of the *nagB *gene within both groups of protists will be needed to solidify such inferences. Finally, the absence of *fprA *in the *Dictyostelium discoideum *genome suggests that the presence of this eubacterial gene within various Amoebozoa lineages might be used as a synapomorphy for discerning phylogenetic relationships within the group.

Similarly, diplomonads and parabasalids have been suggested to share a common ancestor, initially mainly based on weak evidence from molecular data [53,54]. The case for this relationship was recently strengthened by the identification of two aminoacyl-tRNA synthetase genes that appear to have been transferred to a common ancestor of the two lineages [18] and recent phylogenetic analyses of concatenated protein alignments [25-27]. The observation of a transfer of a gene encoding A-type flavoprotein to a uniquely shared ancestor of the two lineages (Figure 3) further supports their specific relationship. The identification of these three genes of prokaryotic origin shared between diplomonads and parabasalids in other lineages within Excavata should be useful to pinpoint relationships within this poorly resolved and diverse group of eukaryotes.

### The timing of transfers relative to eukaryote diversification

**Figure 7:**
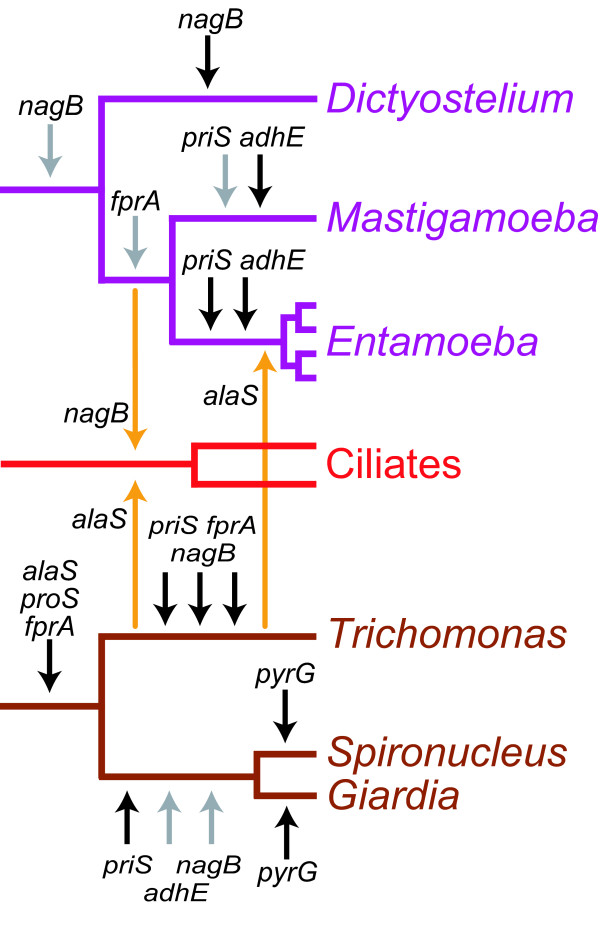
**Summary of putative lateral gene transfers affecting amoebozoa, ciliates, and diplomonads, and parabasalids**. Lateral gene transfers inferred from Figures 2-6, as well as previously published phylogenetic analyses [10, 18] discussed in the text, are indicated on the topology; gene transfers from prokaryotes are indicated by black arrows, intra-eukaryote transfers between the groups are indicated by orange arrows, and gene introduced from uncertain origins are indicated by grey arrow. Please notice that the figure does not delineate the order of individual transfer events on each branch, and that plausible alternative hypotheses do exist to explain some of the unexpected phylogenetic positions of eukaryotes, here indicated as gene transfer events, our currently preferred hypothesis (see text for details).

The relative timing of the transfers can be addressed in more detail with our increased taxon sampling (Figure 7). For reasons outlined above, probably none of the four genes was present in the last common eukaryotic ancestor indicating that all putative transfers almost certainly happened in a more recent evolutionary time (Figure 7). However, all four genes were transferred to the diplomonad lineage before the split between *Giardia *and *Spironucleus *– they branch together in the phylogenetic reconstructions (Figures 2, 3, 4 &6). With the sampling of *Trichomonas *homologs for three of the genes and the absence of the fourth, we now can date the transfers of *priS*, *nagB*, and *adhE *to after the split between diplomonads and parabasalids [18,25-27], but before the divergence of the two major groups of diplomonads [55] (Figure 7). The fourth gene (*fprA*) was most likely introduced into the diplomonads lineage before the split of parabasalids, but after their divergence to the other eukaryotic lineages.

Similarly, the putative transfers previously found to be affecting the *Entamoeba *lineage [10] can be dated in more detail with our updated datasets. The *Entamoeba *sequences branch together in the phylogenetic reconstructions for all genes, indicating that the genes were present in the common ancestor of the four species included in the analysis (Figures 2, 3, 4, 5, 6). For the other two genes, *priS *and *adhE*, the separation of the *Entamoeba *and *Mastigamoeba *sequences is strongly supported (Figures 2 &6), indicating that the transfer event to the *Entamoeba *lineage probably happened after the split between *Entamoeba *and pelobionts, but before the divergence of the *Entamoeba *species [21]. A similar pattern was observed for the gene encoding alanyl-tRNA synthetase, where the ancestral eukaryotic version was replaced by a homolog from the parabasalid lineage in *Entamoeba *after the split from the *Mastigamoeba *[18]. The timing of the transfer of the *priS *gene is more difficult to pinpoint since the separation of the *Entamoeba *and *Mastigamoeba *sequences is only weakly supported by the bootstrap analyses (Figure 2). As mentioned above, *nagB *and *fprA *most likely were present in the common ancestor of *Mastigamoeba *and *Entamoeba*, and the recipient of the *nagB *gene most likely was a common ancestor also of *Dictyostelium*, indicating that the transfer of these genes probably were more ancient events than the transfers of *priS *and *adhE *(Figure 7). The multiple copies found in one or more of the *Entamoeba *species for *priS*, and *fprA *are most likely due to recent gene duplication events within the *Entamoeba *lineages (Figures 2 &3), a pattern also observed from the analysis of the partial genome sequence of *E. invadens*[56]. Interestingly, one of the two *E. invadens priS *sequences has a frameshift due to an eight nucleotide long deletion in the middle of the gene (this is unlikely to be due to a methodological artefact as several different PCR products gave identical sequences). This frameshift probably reflects the dynamics of the evolution of gene families in the *Entamoeba *lineage with frequent gene duplication followed by inactivation of some of the paralogs by accumulation of deleterious mutations.

Among the four sampled genes, the absence of gene transfer occurring within the *Entamoeba *and diplomonad groups is probably an indication that the evolutionary times since the split of these respective groups are short in comparison to the time since the last common eukaryotic ancestor, rather than an indication that the rate of inter-domain transfers have decreased in more recent evolutionary time. Indeed, one of the fifteen genes in the previous analysis, *pyrG *which encodes CTP synthetase, was probably introduced independently into the *Giardia *and *Spironucleus *lineages [10]. However, the data are still scarce, and additional sampling of genes from diverse protist lineages could change the inferences of the timing of the transfers of individual genes presented here (Figure 7). Nevertheless, the data from our four genes, in combination with previously published data [10,18], support a scenario where prokaryotic genes from various lineages have been transferred into eukaryotic lineages continuously over time (Figure 7).

### A link between gene transfers and feeding habits in phagotrophic protists and their shared ecological niche?

An interesting pattern where the studied protists mostly acquire genes from prokaryotes was observed (Figure 7). This may be explained by a preference for growing in prokaryote rich environments and consuming prokaryotes by the four groups of phagotrophic protists investigated in this study – diplomonads, parabasalids, pelobionts and *Entamoeba*, since uptake of DNA from ingested cells is possibly an important mechanism enabling LGT in eukaryotes [4,8]. Indeed, diplomonads generally feed on prokaryotes [57] and several prokaryote-to-eukaryote gene transfer events have been described for this eukaryotic group (Figure 7) [10,41,58,59], while, to our knowledge, no strong case of gene transfer event from a eukaryote lineage to diplomonads has been described yet. *Entamoeba*, on the other hand, is able to ingest both prokaryotes and eukaryotes; it can be maintained in monoxenic cultures with bacteria as well as trypanosomatid flagellates [60,61]. The *Entamoeba *lineage was recently suggested as the recipient lineage in a eukaryote-to-eukaryote gene transfer event of the alanyl-tRNA synthetase gene from the parabasalid lineage [18] (Figure 7), although most gene transfer events affecting *Entamoeba *seem to involve prokaryotic donor lineages (Figures 2, 3, 4, 5, 6) [5,10].

In this study, the donor and recipient lineages could be inferred in one putative eukaryote-to-eukaryote gene transfer event with reasonable support; ciliates were hypothesized to have acquired a gene from an Amoebozoa lineage (Figures 5 and 7). Interestingly, ciliates were also previously shown to represent the recipient lineage in an intra-domain transfer of the alanyl-tRNA synthetase gene [18] (Figure 7). It is possible that the recipient lineage of these two transfers – an ancestor of *Paramecium *and *Tetrahymena *– tended to preferentially graze on eukaryotic protists rather than bacteria and was therefore exposed to eukaryotic DNA leading to LGT events; ciliates are indeed known to eat both prokaryotes and eukaryotes [43,44]. Similarly, dinoflagellates are known to graze on eukaryotes [62] and have been identified as the recipient lineage in eukaryote-to-eukaryote gene transfer events [63,64]. If this pattern holds up in light of more data, it suggests that there is a link between the genome evolution and the food content in phagotrophic protists – indicating that an understanding of eating habits is important to our understanding of gene transfer in the evolution of phagotrophic protists, as postulated by Doolittle [8]. Global proteome phylogenies from 144 prokaryotes indicate that LGT has created pools of shared genes between distantly related prokaryotes occupying the same niche [12], such as mammalian mucosa. Our present analyses extend these observations to microbial eukaryotes with shared genes between microorganisms thriving on mammalian mucosa such as the trichomonads, diplomonads, apicomplexans, *Piromyces *and *Entamoeba*.

## Methods

### Sources of DNA

*Entamoeba invadens *(strain IP-1, ATCC 30994), *E. moshkovskii *(strain FIC, ATCC 30041), *E. terrapinae *(strain M, ATCC 30043) were cultured in LYI-S-2 medium at room temperature [61], *Mastigamoeba balamuthi *(ATCC 30984) were cultured in PYGC medium [65], and *Naegleria gruberi *(strain NEG-M, ATCC 30224) were cultured in modified PYNFH medium (ATCC medium 1034, cat. no. 327-X) at room temperature. The cells were harvested, followed by lysis in 0.25% SDS/0.1 M EDTA, pH 8.0, and genomic DNAs were purified using a cetyl trimethylammonium bromide extraction (CTAB) method [66]. *Trichomonas vaginalis *(strain G3) *nagB *and *fprA *cDNA clones were identified in the ongoing EST project (Hirt, R.P., Embley, T.M., and Harriman, N.), and two *priS Naegleria gruberi *(strain NEG-M, ATCC 30224) cDNA clones were identified in an ongoing EST project (Sjögren, Å.M., Andersson, J.O., Gill, E., Roger, A.J., unpublished).

### PCR and sequencing

Exact match PCR primers to obtain *Entamoeba *genes for independent sequencing were designed based on sequences available from the website of the ongoing genome projects [67] that showed similarity to the studied genes. If no such sequences were available, degenerate primers were designed against conserved regions of the alignments. Using different combinations of these primers the four genes were successfully amplified using genomic DNA in PCR reactions. The *Mastigamoeba priS *sequence was amplified using degenerate primers and genomic DNA, while the *Mastigamoeba nagB *and *fprA *were PCR amplified from genomic DNA using exact-match primers designed from cDNA sequences available from an in-house EST project and published [23] cDNA projects, respectively. The *N. gruberi priS *sequence was amplified from genomic DNA using exact match PCR primers based on cDNA sequences. The PCR products were purified using the Qiaquick PCR Purification Kit (Qiagen Inc., Valencia, CA) and directly sequenced using the ABI PRISM BigDye Termination Cycle Sequencing Kit (Applied Biosystems, Foster City, CA) using the primers used in the amplification as well as internal primers. Introns were identified and removed before the subsequent phylogenetic analyses from the obtained *M. balamuthi nagB*, *priS*, and *fprA *sequences.

### Assembly of the datasets

All available homologs for the four genes were retrieved from the National Center for Biotechnology Information [68]. In addition, similarity searches against sequences released from ongoing eukaryotic genome projects were performed to identify and retrieve additional eukaryotic homologs of the genes from ongoing genome projects at various genome sequences centres. Additional *priS*, and *fprA T. vaginalis *sequences, and *nagB *sequences from *Tetrahymena thermophila*, *Trypanosoma. bruci*, and *Trypanosoma cruzi *were retrieved from the Institute for Genomic Research [69]. *Chlamydomonas reinhardtii priS *and *adhE *sequences, and a *Thalassiosira pseudonana priS *sequence were retrieved from the DOE Joint Genome Institute [70]. Finally, a *nagB *sequences from *Leishmania major *and *Paramecium tetraurelia *were retrieved from the Wellcome Trust Sanger Institute [71], and Genoscope [72], respectively. None of the four genes could be detected among the available *Phytophthora sojae *sequences [70].

The aa sequence datasets were aligned using CLUSTALW [73], manually adjusted, and visually inspected to identify unambiguously aligned regions suitable for phylogenetic reconstructions. Only one sequence among pairs with >95% aa sequence identity within the unambiguously aligned regions were retained for further analyses. Finally, all sequences that covered less than one third of the unambiguously aligned regions were excluded. The accessions numbers and other details of the sequences within the datasets are listed in Additional File 1, and the alignments used in the study are available as Additional Files 6, 7, 8, 9. In the previously analyses data from unpublished prokaryotic genome projects were included [10], while only published prokaryotic sequences were included in the present analyses. Therefore, the *Carboxydothermus *sequence and the single *Clostridium *sequence, and the *Carboxydothermus *and *Fibrobacter *sequences are present in the previous *priS *and *fprA *analyses, respectively, but missing from the current datasets.

### Phylogenetic analyses

The optimal aa substitution model for each dataset was selected using the program ModelGenerator, recently developed by T. M. Keane, T. J. Naughton, and J. O. McInerney, National University of Ireland, Maynooth, Ireland [35]. Protein maximum likelihood (ML) phylogenies were inferred using PHYML, version 2.4.4 [34] with the optimal substitution model, and bootstrap support values were calculated based on 500 resampled datasets. Most currently available phylogenetic methods cannot deal with strong aa compositional heterogeneity in the data [74], which may lead to false interpretations of evolutionary events[2,14]. Therefore, we also performed phylogenetic analyses using an approach that ameliorates (or mitigates) the compositional heterogeneity, as recently described in the analyses of the NuoF protein [14]. The aa alignments were recoded into six categories corresponding to the PAM matrix (and most other matrices) as follows: (1) ASTGP, (2) DNEQ, (3) RKH, (4) MVIL, (5) FYW and (6) C [32]. Bayesian phylogenetic analyses were performed on these grouped aa alignments using the program p4 [36]. This allowed the use of a 6 × 6 general time-reversible rate matrix with free parameters rather than a fixed empirical matrix. The among-site rate variation (ASRV) was chosen by the Akaike Information Criterion (AIC) based on ML on the neighbor-joining tree. All parameters, including the composition and substitution rate matrix, were free, and the analysis used the Metropolis-coupled MCMC strategy from MrBayes [75]. Runs were done in duplicate for 10^6 ^generations. The first halves were discarded as burn-in, while the second halves of both runs were combined for calculating the consensus trees. Convergence was assessed by plotting the split support values >0.10 in the two independent runs against each other (Additional File 3). One dataset (the short version of glucosamine-6-phosophate) did not converge well (data not shown) and was rerun in duplicate for 2 × 10^6 ^generations, and the analyses converged under these conditions (Additional File 3). The model fit of the composition was assessed using different approaches. Posterior predictive simulations were performed on the grouped aa datasets [36]. If the tail area probabilities (*p*_*t*_) is low (<0.05), the model composition does not fit the composition of the dataset. Also, tests for compositional homogeneity using *X*^2 ^statistics were performed for both the original non-grouped and the grouped aa datasets using simulations to get the expected null distribution from the obtained trees and preferred substitution models used in the analyses [36]. The more widely used *X*^2^-tests for compositional homogeneity using the χ^2 ^curve as the null distributions are only included for comparison since they fail to take the tree-based correlation of compositions among taxa into account [36] (Additional File 2).

### Nucleotide sequence accession numbers

The sequences reported here were deposited in GenBank under the accession numbers AJ864541-AJ864560.

## Authors' contributions

JOA carried out the molecular biology studies, most bioinformatic analyses, and ML phylogenetic analyses and drafted the manuscript. PGF performed the grouped aa Bayesian phylogenetic analyses. RPH provided two cDNA clones and carried out the analyses for additional file 4. RPH and AJR provided advice on analyses and edited the manuscript. All authors read and approved the final manuscript.

## Supplementary Material

Additional File 1 Lists accession numbers, keys to short names used in alignment files, taxonomic descriptions, and the basis for exclusion from the phylogenetic analyses for all datasets used in the study. Click here for file

Additional File 2 Information about the datasets and parameters of the phylogenetic analyses. Click here for file

Additional File 3 Figures showing the split support for the two runs in the grouped aa analyses plotted one against the other as indicators of convergence. Click here for file

Additional File 4 Figure showing the structural organization of the *T. vaginalis *A-type flavoproteins together with additional discussion. Click here for file

Additional File 5 Figure showing a phylogenetic analysis of the short version of glucosamine-6-phosphate isomerase with the long version and some prokaryotic long branches excluded. Click here for file

Additional File 6 Alignment file in nexus format for the hybrid-cluster protein. Click here for file

Additional File 7 Alignment file in nexus format for the A-type flavoprotein. Click here for file

Additional File 8 Alignment file in nexus format for the glucosamine-6-phosphate isomerase. Click here for file

Additional File 9 Alignment file in nexus format for the alcohol dehydrogenase E. Click here for file
