# Supplementary material for: Evolution of four gene families with patchy phylogenetic distributions: influx of genes into protist genomes
Source: BMC Evol Biol. 2006 Mar 21;6:27. doi: 10.1186/1471-2148-6-27 (PMC1484493; doi:10.1186/1471-2148-6-27)
Supplement: Additional File 1 — Lists accession numbers, keys to short names used in alignment files, taxonomic descriptions, and the basis for exclusion from the phylogenetic analyses for all datasets used in the study. [file 1471-2148-6-27-S1.pdf]

Additional File 1 – Andersson *et al.*

The hybrid-cluster protein dataset.

gi	Short	*Species*	Domain	Group	Comments
32470344	Aci.fer	*Acidithiobacillus ferrooxidans*	B	γ-Proteobacteria	
32033384	Act.ple	*Actinobacillus pleuropneumoniae* serovar 1 str. 4074	B	γ-Proteobacteria	
53765312	Ana.var	*Anabaena variabilis* ATCC 29413	B	Cyanobacteria	
30263400	Bac.ant	*Bacillus anthracis* str. Ames	B	Low G+C Gram positive	
30021542	Bac.cer	*Bacillus cereus* ATCC 14579	B	Low G+C Gram positive	97,1% identity with Bac.ant
53713436	Bac.fra	*Bacteroides fragilis* YCH46	B	Bacteroidetes/Chlorobi group	
29346097	Bac.the	*Bacteroides thetaiotaomicron* VPI-5482	B	Bacteroidetes/Chlorobi group	
46312750	Bur.cep	*Burkholderia cepacia* R18194	B	β-Proteobacteria	
JGI	Chl.re1	*Chlamydomonas reinhardtii*	E	Green algae	
JGI	Chl.re2	*Chlamydomonas reinhardtii*	E	Green algae	
21674516	Chl.tep	*Chlorobium tepidum* TLS	B	Bacteroidetes/Chlorobi group	
15896007	Clo.ac1	*Clostridium acetobutylicum*	B	Low G+C Gram positive	
15896669	Clo.ac2	*Clostridium acetobutylicum*	B	Low G+C Gram positive	
17976847	Clo.pe1	*Clostridium perfringens*	B	Low G+C Gram positive	
18311566	Clo.pe2	*Clostridium perfringens*	B	Low G+C Gram positive	97,6% identity with Clo.pe1
28210163	Clo.tet	*Clostridium tetani* E88	B	Low G+C Gram positive	
23021132	Clo.the	*Clostridium thermocellum* ATCC 27405	B	Low G+C Gram positive	
23111958	Des.haf	*Desulfitobacterium hafniense*	B	Low G+C Gram positive	
50875631	Des.ps1	*Desulfotalea psychrophila* LSv54	B	δ-Proteobacteria	
50876115	Des.ps2	*Desulfotalea psychrophila* LSv54	B	δ-Proteobacteria	
50876114	Des.ps3	*Desulfotalea psychrophila* LSv54	B	δ-Proteobacteria	
20663611	Des.de1	*Desulfovibrio desulfuricans*	B	δ-Proteobacteria	
20663615	Des.de2	*Desulfovibrio desulfuricans*	B	δ-Proteobacteria	100,0% identity with Des.de1
400847	Des.de3	*Desulfovibrio desulfuricans*	B	δ-Proteobacteria	100,0% identity with Des.de1
23474024	Des.de4	*Desulfovibrio desulfuricans* G20	B	δ-Proteobacteria	
46580947	Des.vH1	*Desulfovibrio vulgaris* subsp. vulgaris str. Hildenborough	B	δ-Proteobacteria	
46580421	Des.vH2	*Desulfovibrio vulgaris* subsp. vulgaris str. Hildenborough	B	δ-Proteobacteria	
18252516	Ent.his	*Entamoeba histolytica*	E	Entamoebidae	
this study	Ent.in1	*Entamoeba invadens*	E	Entamoebidae	frameshift
this study	Ent.in2	*Entamoeba invadens*	E	Entamoebidae	
this study	Ent.mos	*Entamoeba moshkovskii*	E	Entamoebidae	
this study	Ent.ter	*Entamoeba terrapinae*	E	Entamoebidae	fragment missing 272 of 418 aligned positions - 80,1% identity with Ent.his
49612112	Erw.car	*Erwinia carotovora* subsp. atroseptica SCRI1043	B	γ-Proteobacteria	
1787099	Esc.co1	*Escherichia coli* K12	B	γ-Proteobacteria	
33347490	Esc.co2	*Escherichia coli* K12	B	γ-Proteobacteria	100,0% identity with Esc.co1
4062452	Esc.co3	*Escherichia coli* K12	B	γ-Proteobacteria	100,0% identity with Esc.co1
19704019	Fus.nuc	*Fusobacterium nucleatum* subsp. nucleatum ATCC 25586	B	Fusobacteria	
48844072	Geo.met	*Geobacter metallireducens* GS-15	B	δ-Proteobacteria	
39995780	Geo.sul	*Geobacter sulfurreducens* PCA	B	δ-Proteobacteria	96,2% identity with Geo.met
18252518	Gia.lam	*Giardia intestinalis*	E	Diplomonad	
48832063	Mag.sp.	*Magnetococcus* sp. MC-1	B	Proteobacteria (unclassified)	
52306398	Man.suc	*Mannheimia succiniciproducens* MBEL55E	B	γ-Proteobacteria	95,7% identity with Act.ple
this study	Mas.bal	*Mastigamoeba balamuthi*	E	Pelobiont	
15668946	Met.ja1	*Methanocaldococcus jannaschii*	A	Euryarchaeota	
7397414	Met.ja2	*Methanocaldococcus jannaschii*	A	Euryarchaeota	100,0% identity with Met.ja1
46142247	Met.bu1	*Methanococcoides burtonii* DSM 6242	A	Euryarchaeota	
53731535	Met.bu2	*Methanococcoides burtonii* DSM 6242	A	Euryarchaeota	
45358283	Met.mar	*Methanococcus maripaludis* S2	A	Euryarchaeota	
21229315	Met.maz	*Methanosarcina mazei* Goe1	A	Euryarchaeota	
7404412	Met.the	*Methanothermobacter thermautotrophicus* str. Delta H	A	Euryarchaeota	
49235681	Mor.the	*Moorella thermoacetica*	B	Low G+C Gram positive	
this study	Nae.gru	*Naegleria gruberi*	E	Heterolobosea	
26522739	Pho.pho	*Photobacterium phosphoreum*	B	γ-Proteobacteria	
34540658	Por.gin	*Porphyromonas gingivalis* W83	B	Bacteroidetes/Chlorobi group	
14521200	Pyr.aby	*Pyrococcus abyssi*	A	Euryarchaeota	
18977064	Pyr.fur	*Pyrococcus furiosus* DSM 3638	A	Euryarchaeota	
33415324	Rho.cap	*Rhodobacter capsulatus*	B	α-Proteobacteria	
22966189	Rho.rub	*Rhodospirillum rubrum*	B	α-Proteobacteria	
16759810	Sal.ent	*Salmonella enterica* subsp. enterica	B	γ-Proteobacteria	96,4% identity with Esc.co1

		serovar Typhi			
16764299	Sal.typ	*Salmonella typhimurium* LT2	B	γ-Proteobacteria	96,6% identity with Esc.co1
24372941	She.one	*Shewanella oneidensis* MR-1	B	γ-Proteobacteria	
24112244	Shi.fle	*Shigella flexneri* 2a str. 301	B	γ-Proteobacteria	99,5% identity with Esc.co1
27983818	Spi.bar	*Spironucleus barkhanus*	E	Diplomonad	
51892166	Sym.the	*Symbiobacterium thermophilum* IAM 14863	B	High G+C Gram positive	
JGI	Tha.pse	*Thalassiosira pseudonana*	E	Diatom	
20809101	The.ten	*Thermoanaerobacter tengcongensis*	B	Low G+C Gram positive	
15643928	The.mar	*Thermotoga maritima*	B	Thermotogales	
42525995	Tre.den	*Treponema denticola* ATCC 35405	B	Spirochete	
18252520	Tri.va1	*Trichomonas vaginalis*	E	Parabasalid	
TIGR	Tri.va2	*Trichomonas vaginalis*	E	Parabasalid	
59714046	Vib.fis	*Vibrio fischeri* ES114	B	γ-Proteobacteria	
28897963	Vib.par	*Vibrio parahaemolyticus* RIMD 2210633	B	γ-Proteobacteria	
27366144	Vib.vul	*Vibrio vulnificus* CMCP6	B	γ-Proteobacteria	96,6% identity with Vib.par
34557553	Wol.suc	*Wolinella succinogenes*	B	ε-Proteobacteria	
22126696	Yer.pes	*Yersinia pestis* KIM	B	γ-Proteobacteria	
56542587	Zym.mob	*Zymomonas mobilis* subsp. mobilis ZM4	B	α-Proteobacteria	

The A-type flavoprotein dataset.

gi	Short	Species	Domain	Group	Comment
37913107	Aer.hyd	*Aeromonas hydrophila*	B	γ-Proteobacteria	fragment missing 120 of 269 aligned postions – 91,5% identical with Erw.car
45510255	Ana.va1	*Anabaena variabilis* ATCC 29413	B	Cyanobacteria	99,3% identity with Nos.sp5
53764133	Ana.va2	*Anabaena variabilis* ATCC 29413	B	Cyanobacteria	96,3% identity with Nos.sp3
53764449	Ana.va3	*Anabaena variabilis* ATCC 29413	B	Cyanobacteria	97,8% identity with Nos.sp1
53764450	Ana.va4	*Anabaena variabilis* ATCC 29413	B	Cyanobacteria	100,0% identity with Nos.sp2
45510253	Ana.va5	*Anabaena variabilis* ATCC 29413	B	Cyanobacteria	97,0% identity with Nos.sp6
53764134	Ana.va6	*Anabaena variabilis* ATCC 29413	B	Cyanobacteria	96,3% identity with Nos.sp4
11497784	Arc.fu1	*Archaeoglobus fulgidus* DSM 4304	A	Euryarchaeota	
11499115	Arc.fu2	*Archaeoglobus fulgidus* DSM 4304	A	Euryarchaeota	
56475711	Azo.spE	*Azoarcus* sp. EbN1	B	β-Proteobacteria	
60491887	Bac.fra	*Bacteroides fragilis* NCTC 9343	B	Bacteroidetes/Chlorobi group	
29349534	Bac.the	*Bacteroides thetaiotaomicron* VPI-5482	B	Bacteroidetes/Chlorobi group	
21675091	Chl.tep	*Chlorobium tepidum* TLS	B	Bacteroidetes/Chlorobi group	
15894314	Clo.ac1	*Clostridium acetobutylicum*	B	Low G+C Gram positives	
15895714	Clo.ac2	*Clostridium acetobutylicum*	B	Low G+C Gram positives	
18309270	Clo.pe1	*Clostridium perfringens*	B	Low G+C Gram positives	
18309763	Clo.pe2	*Clostridium perfringens*	B	Low G+C Gram positives	
18309996	Clo.pe3	*Clostridium perfringens*	B	Low G+C Gram positives	
28210710	Clo.te1	*Clostridium tetani* E88	B	Low G+C Gram positives	
28211064	Clo.te2	*Clostridium tetani* E88	B	Low G+C Gram positives	
28211481	Clo.te3	*Clostridium tetani* E88	B	Low G+C Gram positives	
28211510	Clo.te4	*Clostridium tetani* E88	B	Low G+C Gram positives	
23020486	Clo.the	*Clostridium thermocellum* ATCC 27405	B	Low G+C Gram positives	
45528365	Cro.wa1	*Crocosphaera watsonii* WH 8501	B	Cyanobacteria	
45527451	Cro.wa2	*Crocosphaera watsonii* WH 8501	B	Cyanobacteria	
41722689	Dec.aro	*Dechloromonas aromatica* RCB	B	β-Proteobacteria	
50876536	Des.ps1	*Desulfotalea psychrophila* LSv54	B	δ-Proteobacteria	
50877008	Des.ps2	*Desulfotalea psychrophila* LSv54	B	δ-Proteobacteria	
50876535	Des.ps3	*Desulfotalea psychrophila* LSv54	B	δ-Proteobacteria	
23473638	Des.des	*Desulfovibrio desulfuricans* G20	B	δ-Proteobacteria	
14916694	Des.gig	*Desulfovibrio gigas*	B	δ-Proteobacteria	
46581587	Des.vu1	*Desulfovibrio vulgaris* subsp. vulgaris str. Hildenborough	B	δ-Proteobacteria	
46580422	Des.vu2	*Desulfovibrio vulgaris* subsp. vulgaris str. Hildenborough	B	δ-Proteobacteria	
56474182	Ent.hi1	*Entamoeba histolytica*	E	Entamoebidae	
56471042	Ent.hi2	*Entamoeba histolytica*	E	Entamoebidae	
56468393	Ent.hi3	*Entamoeba histolytica*	E	Entamoebidae	
56468595	Ent.hi4	*Entamoeba histolytica*	E	Entamoebidae	
this study	Ent.inv	*Entamoeba invadens*	E	Entamoebidae	
this study	Ent.mo1	*Entamoeba moshkovskii*	E	Entamoebidae	
this study	Ent.mo2	*Entamoeba moshkovskii*	E	Entamoebidae	
this study	Ent.ter	*Entamoeba terrapinae*	E	Entamoebidae	
49610370	Erw.car	*Erwinia carotovora* subsp. atroseptica SCRI1043	B	γ-Proteobacteria	
16130617	Esc.col	*Escherichia coli* K12	B	γ-Proteobacteria	
19703847	Fus.nu1	*Fusobacterium nucleatum* subsp. nucleatum ATCC 25586	B	Fusobacteria	
19704755	Fus.nu2	*Fusobacterium nucleatum* subsp. nucleatum ATCC 25586	B	Fusobacteria	
23055925	Geo.me1	*Geobacter metallireducens*	B	δ-Proteobacteria	
23056906	Geo.me2	*Geobacter metallireducens*	B	δ-Proteobacteria	100,0% identity with Geo.me1
39998383	Geo.sul	*Geobacter sulfurreducens* PCA	B	δ-Proteobacteria	
27981645	Gia.lam	*Giardia intestinalis*	E	Diplomonads	
37521344	Glo.vi1	*Gloeobacter violaceus*	B	Cyanobacteria	
37521345	Glo.vi2	*Gloeobacter violaceus*	B	Cyanobacteria	
this study	Mas.bal	*Mastigamoeba balamuthi*	E	Pelobionts	
46141957	Met.bur	*Methanococcoides burtonii* DSM 6242	A	Euryarchaeota	
15668714	Met.ja1	*Methanococcus jannaschii*	A	Euryarchaeota	
15668913	Met.ja2	*Methanococcus jannaschii*	A	Euryarchaeota	
15668929	Met.ja3	*Methanococcus jannaschii*	A	Euryarchaeota	
2127904	Met.ja4	*Methanococcus jannaschii*	A	Euryarchaeota	100,0% identity with Met.ja1
45047591	Met.ma1	*Methanococcus maripaludis* S2	A	Euryarchaeota	
45047564	Met.ma2	*Methanococcus maripaludis* S2	A	Euryarchaeota	
45047563	Met.ma3	*Methanococcus maripaludis* S2	A	Euryarchaeota	95,9% identity with Met.ma2
20092195	Met.ac1	*Methanosarcina acetivorans* str. C2A	A	Euryarchaeota	
20092541	Met.ac2	*Methanosarcina acetivorans* str. C2A	A	Euryarchaeota	

48837637	Met.bar	*Methanosarcina barkeri* str. fusaro	A	Euryarchaeota	
21229289	Met.maz	*Methanosarcina mazei* Goe1	A	Euryarchaeota	
21362551	Met.mar	*Methanothermobacter marburgensis* str. Marburg	A	Euryarchaeota	
15678185	Met.th1	*Methanothermobacter thermautotrophicus* str. Delta H	A	Euryarchaeota	
15678248	Met.th2	*Methanothermobacter thermautotrophicus* str. Delta H	A	Euryarchaeota	
2127680	Met.th3	*Methanothermobacter thermautotrophicus* str. Delta H	A	Euryarchaeota	
21362560	Moo.the	*Moorella thermoacetica*	B	Low G+C Gram positives	
23123589	Nos.pu1	*Nostoc punctiforme*	B	Cyanobacteria	
23123808	Nos.pu2	*Nostoc punctiforme*	B	Cyanobacteria	
23123810	Nos.pu3	*Nostoc punctiforme*	B	Cyanobacteria	
23123861	Nos.pu4	*Nostoc punctiforme*	B	Cyanobacteria	
23129575	Nos.pu5	*Nostoc punctiforme*	B	Cyanobacteria	
23129576	Nos.pu6	*Nostoc punctiforme*	B	Cyanobacteria	
17227673	Nos.sp1	*Nostoc* sp. PCC 7120	B	Cyanobacteria	
17227674	Nos.sp2	*Nostoc* sp. PCC 7120	B	Cyanobacteria	
17231383	Nos.sp3	*Nostoc* sp. PCC 7120	B	Cyanobacteria	
17231387	Nos.sp4	*Nostoc* sp. PCC 7120	B	Cyanobacteria	
17231936	Nos.sp5	*Nostoc* sp. PCC 7120	B	Cyanobacteria	
17231938	Nos.sp6	*Nostoc* sp. PCC 7120	B	Cyanobacteria	
54308337	Pho.pro	*Photobacterium profundum* SS9	B	γ-Proteobacteria	
34540586	Por.gin	*Porphyromonas gingivalis* W83	B	Bacteroidetes/Chlorobi group	
33864428	Pro.mM1	*Prochlorococcus marinus* str. MIT 9313	B	Cyanobacteria	
33864429	Pro.mM2	*Prochlorococcus marinus* str. MIT 9313	B	Cyanobacteria	
33239496	Pro.mC1	*Prochlorococcus marinus* subsp. marinus str. CCMP1375	B	Cyanobacteria	
33239497	Pro.mC2	*Prochlorococcus marinus* subsp. marinus str. CCMP1375	B	Cyanobacteria	
33860602	Pro.mC3	*Prochlorococcus marinus* subsp. pastoris str. CCMP1378	B	Cyanobacteria	
33860603	Pro.mC4	*Prochlorococcus marinus* subsp. pastoris str. CCMP1378	B	Cyanobacteria	
14521096	Pyr.aby	*Pyrococcus abyssi*	A	Euryarchaeota	
18977066	Pyr.fu1	*Pyrococcus furiosus* DSM 3638	A	Euryarchaeota	
18977123	Pyr.fu2	*Pyrococcus furiosus* DSM 3638	A	Euryarchaeota	
14590919	Pyr.hor	*Pyrococcus horikoshii*	A	Euryarchaeota	
1075539	Rho.ca1	*Rhodobacter capsulatus*	B	α-Proteobacteria	97,0% identity with Rho.ca2
21431763	Rho.ca2	*Rhodobacter capsulatus*	B	α-Proteobacteria	
22958095	Rho.sph	*Rhodobacter sphaeroides*	B	α-Proteobacteria	
16761617	Sal.ent	*Salmonella enterica* subsp. enterica serovar Typhi	B	γ-Proteobacteria	96,3% identity with Esc.col
16766146	Sal.typ	*Salmonella typhimurium* LT2	B	γ-Proteobacteria	96,3% identity with Esc.col
24114007	Shi.fle	*Shigella flexneri* 2a str. 301	B	γ-Proteobacteria	99,3% identity with Esc.col
27984029	Spi.bar	*Spironucleus barkhanus*	E	Diplomonads	
56752293	Syn.el1	*Synechococcus elongatus* PCC 6301	B	Cyanobacteria	
45513282	Syn.el2	*Synechococcus elongatus* PCC 6301	B	Cyanobacteria	100,0% identity with Syn.el1
56752292	Syn.el3	*Synechococcus elongatus* PCC 6301	B	Cyanobacteria	
45513283	Syn.el4	*Synechococcus elongatus* PCC 6301	B	Cyanobacteria	99,6% identity with Syn.el3
33866897	Syn.sW1	*Synechococcus* sp. WH 8102	B	Cyanobacteria	
33866898	Syn.sW2	*Synechococcus* sp. WH 8102	B	Cyanobacteria	
16329320	Syn.sP1	*Synechocystis* sp. PCC 6803	B	Cyanobacteria	
16329322	Syn.sP2	*Synechocystis* sp. PCC 6803	B	Cyanobacteria	
16331061	Syn.sP3	*Synechocystis* sp. PCC 6803	B	Cyanobacteria	
16331685	Syn.sP4	*Synechocystis* sp. PCC 6803	B	Cyanobacteria	
20807480	The.ten	*Thermoanaerobacter tengcongensis*	B	Low G+C Gram positives	
57159073	The.kod	*Thermococcus kodakaraensis* KOD1	A	Euryarchaeota	
22298632	The.el1	*Thermosynechococcus elongatus* BP-1	B	Cyanobacteria	
22298916	The.el2	*Thermosynechococcus elongatus* BP-1	B	Cyanobacteria	
15643518	The.mar	*Thermotoga maritima*	B	Thermotogales	
42526588	Tre.den	*Treponema denticola* ATCC 35405	B	Spirochetes	
23039893	Tri.er1	*Trichodesmium erythraeum* IMS101	B	Cyanobacteria	
23043405	Tri.er2	*Trichodesmium erythraeum* IMS101	B	Cyanobacteria	
this study	Tri.va1	*Trichomonas vaginalis*	E	Parabasalids	
TIGR	Tri.va2	*Trichomonas vaginalis*	E	Parabasalids	
TIGR	Tri.va3	*Trichomonas vaginalis*	E	Parabasalids	
37908704	Tri.va4	*Trichomonas vaginalis*	E	Parabasalids	
59712389	Vib.fis	*Vibrio fischeri* ES114	B	γ-Proteobacteria	
27367713	Vib.vul	*Vibrio vulnificus* CMCP6	B	γ-Proteobacteria	
34556611	Wol.suc	*Wolinella succinogenes*	B	ε-Proteobacteria	

The glucosamine-6-phosphate isomerase dataset.

gi	Short	Species	Domain	Group	Comment
46143414	Act.ple	*Actinobacillus pleuropneumoniae* serovar 1 str. 4074	B	γ-Proteobacteria	
45509250	Ana.var	*Anabaena variabilis* ATCC 29413	B	Cyanobacteria	97,8% identity with Nos.sp
31239371	Ano.gam	*Anopheles gambiae*	E	Metazoa	
66547108	Api.mel	*Apis mellifera*	E	Metazoa	
40745392	Asp.nid	*Aspergillus nidulans* FGSC A4	E	Fungi	
21402108	Bac.ant	*Bacillus anthracis* A2012	B	Low G+C Gram positives	
30022141	Bac.cer	*Bacillus cereus* ATCC 14579	B	Low G+C Gram positives	98,2% identity with Bac.ant
56963255	Bac.cla	*Bacillus clausii* KSM-K16	B	Low G+C Gram positives	
15612983	Bac.hal	*Bacillus halodurans*	B	Low G+C Gram positives	
52079159	Bac.li1	*Bacillus licheniformis* ATCC 14580	B	Low G+C Gram positives	
52082623	Bac.li2	*Bacillus licheniformis* ATCC 14580	B	Low G+C Gram positives	
31076788	Bac.sph	*Bacillus sphaericus*	B	Low G+C Gram positives	
16077305	Bac.su1	*Bacillus subtilis*	B	Low G+C Gram positives	
16080555	Bac.su2	*Bacillus subtilis*	B	Low G+C Gram positives	
60493857	Bac.fr1	*Bacteroides fragilis* NCTC 9343	B	Bacteroidetes/Chlorobi group	
60491888	Bac.fr2	*Bacteroides fragilis* NCTC 9343	B	Bacteroidetes/Chlorobi group	96,9% identity with Bac.th3
29345668	Bac.th1	*Bacteroides thetaiotaomicron* VPI-5482	B	Bacteroidetes/Chlorobi group	long
29348995	Bac.th2	*Bacteroides thetaiotaomicron* VPI-5482	B	Bacteroidetes/Chlorobi group	
29349535	Bac.th3	*Bacteroides thetaiotaomicron* VPI-5482	B	Bacteroidetes/Chlorobi group	
23335426	Bif.lon	*Bifidobacterium longum* DJO10A	B	High G+C Gram positives	
33519783	Blo.flo	*Blochmannia floridanus*	B	γ-Proteobacteria	
15594497	Bor.bur	*Borrelia burgdorferi*	B	Spirochetes	
51572983	Bor.gar	*Borrelia garinii* PBi	B	Spirochetes	
61811995	Bos.tau	*Bos taurus*	E	Metazoa	98,3% identity with Hom.sa1
62422909	Bre.lin	*Brevibacterium linens* BL2	B	High G+C Gram positives	
39586581	Cae.bri	*Caenorhabditis briggsae*	E	Metazoa	
17554876	Cae.ele	*Caenorhabditis elegans*	E	Metazoa	
13702164	Can.al1	*Candida albicans*	E	Fungi	
417340	Can.al2	*Candida albicans*	E	Fungi	99,6% identity with Can.al1
57042718	Can.fam	*Canis familiaris*	E	Metazoa	96,1% identity with Hom.sa1
15893480	Clo.ace	*Clostridium acetobutylicum*	B	Low G+C Gram positives	
18311416	Clo.per	*Clostridium perfringens*	B	Low G+C Gram positives	
28212200	Clo.tet	*Clostridium tetani* E88	B	Low G+C Gram positives	
38233130	Cor.dip	*Corynebacterium diphtheriae* NCTC 13129	B	High G+C Gram positives	
25029074	Cor.eff	*Corynebacterium efficiens* YS-314	B	High G+C Gram positives	
19553842	Cor.glu	*Corynebacterium glutamicum* ATCC 13032	B	High G+C Gram positives	
45522698	Cro.wat	*Crocosphaera watsonii* WH 8501	B	Cyanobacteria	
50254779	Cry.neo	*Cryptococcus neoformans* var. neoformans B-3501A	E	Fungi	
23135910	Cyt.hut	*Cytophaga hutchinsonii*	B	Bacteroidetes/Chlorobi group	long
62202396	Dan.rer	*Danio rerio*	E	Metazoa	
49657079	Deb.han	*Debaryomyces hansenii* CBS767	E	Fungi	
23112797	Des.haf	*Desulfitobacterium hafniense*	B	Low G+C Gram positives	
60466211	Dic.di1	*Dictyostelium discoideum*	E	Mycetozoa	long
60470057	Dic.di2	*Dictyostelium discoideum*	E	Mycetozoa	
19920764	Dro.mel	*Drosophila melanogaster*	E	Metazoa	
56470258	Ent.his	*Entamoeba histolytica*	E	Entamoebidae	long
this study	Ent.inv	*Entamoeba invadens*	E	Entamoebidae	long
this study	Ent.mos	*Entamoeba moshkovskii*	E	Entamoebidae	long
this study	Ent.ter	*Entamoeba terrapinae*	E	Entamoebidae	long
29375097	Ent.fa2	*Enterococcus faecalis* V583	B	Low G+C Gram positives	
22990925	Ent.fa1	*Enterococcus faecium*	B	Low G+C Gram positives	
49610791	Erw.car	*Erwinia carotovora* subsp. atroseptica SCRI1043	B	γ-Proteobacteria	
20150540	Esc.co2	*Escherichia coli*	B	γ-Proteobacteria	
3005594	Esc.co3	*Escherichia coli*	B	γ-Proteobacteria	100,0% identity with Esc.co2
16131033	Esc.co1	*Escherichia coli* K12	B	γ-Proteobacteria	
46113349	Exi.sp2	*Exiguobacterium* sp. 255-15	B	Low G+C Gram positives	
19704478	Fus.nuc	*Fusobacterium nucleatum* subsp. nucleatum ATCC 25586	B	Fusobacteria	
50747027	Gal.ga1	*Gallus gallus*	E	Metazoa	95,2% identity with Hom.sa2
57525040	Gal.ga2	*Gallus gallus*	E	Metazoa	
53130328	Gal.ga3	*Gallus gallus*	E	Metazoa	100,0% identity with Gal.ga2

56420811	Geo.kau	*Geobacillus kaustophilus* HTA426	B	Low G+C Gram positives	
6831555	Gia.la1	*Giardia lamblia*	E	Diplomonads	
6831556	Gia.la2	*Giardia lamblia*	E	Diplomonads	
6090573	Gia.la3	*Giardia lamblia*	E	Diplomonads	99,1% identity with Gia.la3
42548307	Gib.ze1	*Gibberella zeae* PH-1	E	Fungi	
42555043	Gib.ze2	*Gibberella zeae* PH-1	E	Fungi	
33152849	Hae.duc	*Haemophilus ducreyi* 35000HP	B	γ-Proteobacteria	
16272108	Hae.inf	*Haemophilus influenzae* Rd	B	γ-Proteobacteria	
32030023	Hae.som	*Haemophilus somnus* 2336	B	γ-Proteobacteria	
13027378	Hom.sa1	*Homo sapiens*	E	Metazoa	
15930199	Hom.sa2	*Homo sapiens*	E	Metazoa	
16550419	Hom.sa3	*Homo sapiens*	E	Metazoa	98,7% identity with Hom.sa2
19923881	Hom.sa4	*Homo sapiens*	E	Metazoa	99,1% identity with Hom.sa2
58338189	Lac.aci	*Lactobacillus acidophilus* NCFM	B	Low G+C Gram positives	
62513852	Lac.cas	*Lactobacillus casei* ATCC 334	B	Low G+C Gram positives	
23003825	Lac.gas	*Lactobacillus gasseri*	B	Low G+C Gram positives	
42519872	Lac.joh	*Lactobacillus johnsonii* NCC 533	B	Low G+C Gram positives	99,1% identity with Lac.gas
28377158	Lac.pla	*Lactobacillus plantarum* WCFS1	B	Low G+C Gram positives	
15673555	Lac.lac	*Lactococcus lactis* subsp. lactis	B	Low G+C Gram positives	
50955485	Lei.xyl	*Leifsonia xyli* subsp. xyli str. CTCB07	B	High G+C Gram positives	
Sanger	Lei.maj	*Leishmania major*	E	Euglenozoa	
23024189	Leu.mes	*Leuconostoc mesenteroides* subsp. mesenteroides ATCC 8293	B	Low G+C Gram positives	
16799949	Lis.in1	*Listeria innocua*	B	Low G+C Gram positives	
16800025	Lis.in2	*Listeria innocua*	B	Low G+C Gram positives	98,7% identity with Lis.mo2
16801514	Lis.in3	*Listeria innocua*	B	Low G+C Gram positives	
16802918	Lis.mo1	*Listeria monocytogenes* EGD-e	B	Low G+C Gram positives	
16802997	Lis.mo2	*Listeria monocytogenes* EGD-e	B	Low G+C Gram positives	
16804396	Lis.mo3	*Listeria monocytogenes* EGD-e	B	Low G+C Gram positives	
38102084	Mag.gri	*Magnaporthe grisea* 70-15	E	Fungi	
23012890	Mag.mag	*Magnetospirillum magnetotacticum*	B	α-Proteobacteria	
52308312	Man.suc	*Mannheimia succiniciproducens* MBEL55E	B	γ-Proteobacteria	
this study	Mas.bal	*Mastigamoeba balamuthi*	E	Pelobionts	fragment missing 128 of 229 aligned postions – 54,4% identity with Ent.inv; long version missing 438 of 562 aligned positions
3122434	Mes.aur	*Mesocricetus auratus*	E	Metazoa	96,9% identity with Hom.sa1
45914196	Mes.spB	*Mesorhizobium* sp. BNC1	B	α-Proteobacteria	
12644084	Mus.mu1	*Mus musculus*	E	Metazoa	96,9% identity with Hom.sa1
12853907	Mus.mu2	*Mus musculus*	E	Metazoa	97,4% identity with Hom.sa2
19882233	Mus.mu3	*Mus musculus*	E	Metazoa	97,4% identity with Hom.sa2
5353761	Mus.mu4	*Mus musculus*	E	Metazoa	97,4% identity with Hom.sa1
6754030	Mus.mu5	*Mus musculus*	E	Metazoa	96,1% identity with Hom.sa1
54020607	Myc.hyo	*Mycoplasma hyopneumoniae* 232	B	Low G+C Gram positives	
42561343	Myc.my1	*Mycoplasma mycoides* subsp. mycoides SC str. PG1	B	Low G+C Gram positives	
42560683	Myc.my2	*Mycoplasma mycoides* subsp. mycoides SC str. PG1	B	Low G+C Gram positives	
26553629	Myc.pen	*Mycoplasma penetrans*	B	Low G+C Gram positives	
15828833	Myc.pul	*Mycoplasma pulmonis*	B	Low G+C Gram positives	
32406955	Neu.cra	*Neurospora crassa*	E	Fungi	
23127811	Nos.pun	*Nostoc punctiforme*	B	Cyanobacteria	
17228222	Nos.sp	*Nostoc* sp. PCC 7120	B	Cyanobacteria	
23098066	Oce.ihe	*Oceanobacillus iheyensis* HTE831	B	Low G+C Gram positives	
23038068	Oen.oen	*Oenococcus oeni* MCW	B	Low G+C Gram positives	
Genoscope	Par.tet	*Paramecium tetraurelia*	E	Ciliates	long
15602740	Pas.mul	*Pasteurella multocida*	B	γ-Proteobacteria	
48870313	Ped.pen	*Pediococcus pentosaceus* ATCC 25745	B	Low G+C Gram positives	
54302040	Pho.pr1	*Photobacterium profundum* SS9	B	γ-Proteobacteria	
54308224	Pho.pr2	*Photobacterium profundum* SS9	B	γ-Proteobacteria	
37525283	Pho.lum	*Photorhabdus luminescens* subsp. laumondii TTO1	B	γ-Proteobacteria	
55730197	Pon.pyg	*Pongo pygmaeus*	E	Metazoa	99,6% identity with Hom.sa1
34540585	Por.gi1	*Porphyromonas gingivalis* W83	B	Bacteroidetes/Chlorobi group	
34540986	Por.gi2	*Porphyromonas gingivalis* W83	B	Bacteroidetes/Chlorobi group	long
33863691	Pro.mar	*Prochlorococcus marinus* str. MIT 9313	B	Cyanobacteria	
50841959	Pro.acn	*Propionibacterium acnes* KPA171202	B	High G+C Gram positives	
27695235	Rat.nor	*Rattus norvegicus*	E	Metazoa	97,8% identity with Hom.sa2

32471377	Rho.ba1	*Rhodopirellula baltica* SH 1	B	Planctomycetes	
32472019	Rho.ba2	*Rhodopirellula baltica* SH 1	B	Planctomycetes	
32472456	Rho.ba3	*Rhodopirellula baltica* SH 1	B	Planctomycetes	
32472581	Rho.ba4	*Rhodopirellula baltica* SH 1	B	Planctomycetes	long
46107074	Rub.xyl	*Rubrobacter xylanophilus* DSM 9941	B	High G+C Gram positives	
16759628	Sal.ent	*Salmonella enterica* subsp. enterica serovar Typhi	B	γ-Proteobacteria	95,6% identity with Esc.co2
16764054	Sal.typ	*Salmonella typhimurium* LT2	B	γ-Proteobacteria	95,6% identity with Esc.co2
56756765	Sch.jap	*Schistosoma japonicum*	E	Metazoa	
24112036	Shi.fl1	*Shigella flexneri* 2a str. 301	B	γ-Proteobacteria	99,6% identity with Esc.co2
24114429	Shi.fl2	*Shigella flexneri* 2a str. 301	B	γ-Proteobacteria	98,6% identity with Esc.co1
27982841	Spi.ba1	*Spironucleus barkhanus*	E	Diplomonads	fragment missing 107 of 229 aligned postions – 68,0% identity with Spi.ba2
27983038	Spi.ba2	*Spironucleus barkhanus*	E	Diplomonads	
15923559	Sta.aur	*Staphylococcus aureus* subsp. aureus Mu50	B	Low G+C Gram positives	
27467258	Sta.epi	*Staphylococcus epidermidis* ATCC 12228	B	Low G+C Gram positives	
22536963	Str.aga	*Streptococcus agalactiae* 2603V/R	B	Low G+C Gram positives	
24379109	Str.mut	*Streptococcus mutans* UA159	B	Low G+C Gram positives	
15901269	Str.pne	*Streptococcus pneumoniae* TIGR4	B	Low G+C Gram positives	
15675320	Str.pyo	*Streptococcus pyogenes*	B	Low G+C Gram positives	
50591240	Str.sui	*Streptococcus suis* 89/1591	B	Low G+C Gram positives	
62526838	Str.the	*Streptococcus thermophilus* LMD-9	B	Low G+C Gram positives	
29829560	Str.ave	*Streptomyces avermitilis* MA-4680	B	High G+C Gram positives	
21223604	Str.coe	*Streptomyces coelicolor* A3(2)	B	High G+C Gram positives	
51891744	Sym.the	*Symbiobacterium thermophilum* IAM 14863	B	High G+C Gram positives	
56750455	Syn.elo	*Synechococcus elongatus* PCC 6301	B	Cyanobacteria	
33865076	Syn.sp	*Synechococcus* sp. WH 8102	B	Cyanobacteria	
TIGR	Tet.the	*Tetrahymena thermophila*	E	Ciliates	long
47222915	Tet.nig	*Tetraodon nigroviridis*	E	Metazoa	
20807502	The.ten	*Thermoanaerobacter tengcongensis*	B	Low G+C Gram positives	
42525853	Tre.den	*Treponema denticola* ATCC 35405	B	Spirochetes	
23043712	Tri.ery	*Trichodesmium erythraeum* IMS101	B	Cyanobacteria	
this study	Tri.vag	*Trichomonas vaginalis*	E	Parabasalids	long
TIGR	Try.bru	*Trypanosoma brucei*	E	Euglenozoa	
TIGR	Try.cru	*Trypanosoma cruzi*	E	Euglenozoa	
46097301	Usi.may	*Ustilago maydis* 521	E	Fungi	
15601778	Vib.cho	*Vibrio cholerae* O1 biovar eltor str. N16961	B	γ-Proteobacteria	
59712964	Vib.fis	*Vibrio fischeri* ES114	B	γ-Proteobacteria	
28899893	Vib.par	*Vibrio parahaemolyticus* RIMD 2210633	B	γ-Proteobacteria	
27367580	Vib.vul	*Vibrio vulnificus* CMCP6	B	γ-Proteobacteria	
38014747	Xen.la1	*Xenopus laevis*	E	Metazoa	
38014417	Xen.la2	*Xenopus laevis*	E	Metazoa	
49647197	Yar.lip	*Yarrowia lipolytica* CLIB99	E	Fungi	
16122840	Yer.pes	*Yersinia pestis*	B	γ-Proteobacteria	
51595466	Yer.pse	*Yersinia pseudotuberculosis* IP 32953	B	γ-Proteobacteria	100,0% identity with Yer.pes

The alocohol dehydrogenase E dataset.

gi	Short	Species	Domain	Group	Comment
46143771	Act.ple	*Actinobacillus pleuropneumoniae* serovar 1 str. 4074	B	γ-Proteobacteria	
30264441	Bac.ant	*Bacillus anthracis str. Ames*	B	Low G+C Gram positives	97,0% identity with Bac.cer
30022446	Bac.cer	*Bacillus cereus* ATCC 14579	B	Low G+C Gram positives	
52005783	Bac.lic	*Bacillus licheniformis* ATCC 14580	B	Low G+C Gram positives	
49476825	Bac.thu	*Bacillus thuringiensis* serovar konkukian str. 97-27	B	Low G+C Gram positives	96,9% identity with Bac.thu
23336499	Bif.lon	*Bifidobacterium longum* DJO10A	B	High G+C Gram positives	
JGI	Chl.rei	*Chlamydomonas reinhardtii*	E	Green algae	
34496592	Chr.vio	*Chromobacterium violaceum* ATCC 12472	B	β-Proteobacteria	
15004739	Clo.ac1	*Clostridium acetobutylicum*	B	Low G+C Gram positives	
15004865	Clo.ac2	*Clostridium acetobutylicum*	B	Low G+C Gram positives	
18311513	Clo.per	*Clostridium perfringens*	B	Low G+C Gram positives	
28211045	Clo.tet	*Clostridium tetani* E88	B	Low G+C Gram positives	
23020357	Clo.the	*Clostridium thermocellum* ATCC 27405	B	Low G+C Gram positives	
54659250	Cry.hom	*Cryptosporidium hominis*	E	Apicomplexa	100,0% identity with Cry.par
46228815	Cry.par	*Cryptosporidium parvum*	E	Apicomplexa	
56473007	Ent.hi1	*Entamoeba histolytica* HM-1:IMSS	E	Entamoebidae	
56467638	Ent.hi2	*Entamoeba histolytica* HM-1:IMSS	E	Entamoebidae	99,9% identity with Ent.hi1
56465626	Ent.hi3	*Entamoeba histolytica* HM-1:IMSS	E	Entamoebidae	99,9% identity with Ent.hi1
56472980	Ent.hi4	*Entamoeba histolytica* HM-1:IMSS	E	Entamoebidae	99,9% identity with Ent.hi1
this study	Ent.inv	*Entamoeba invadens*	E	Entamoebidae	
this study	Ent.mos	*Entamoeba moshkovskii*	E	Entamoebidae	
this study	Ent.ter	*Entamoeba terrapinae*	E	Entamoebidae	
29375484	Ent.fas	*Enterococcus faecalis* V583	B	Low G+C Gram positives	
22992835	Ent.fam	*Enterococcus faecium*	B	Low G+C Gram positives	
49611780	Erw.car	*Erwinia carotovora* subsp. atroseptica SCRI1043	B	γ-Proteobacteria	
15801467	Esc.col	*Escherichia coli* O157:H7 EDL933	B	γ-Proteobacteria	
45531125	Exi.sp2	*Exiguobacterium* sp. 255-15	B	Low G+C Gram positives	
2052472	Gia.la1	*Giardia intestinalis*	E	Diplomonads	
29247272	Gia.la2	*Giardia lamblia* ATCC 50803	E	Diplomonads	97,3% identity with Gia.la1
58336794	Lac.aci	*Lactobacillus acidophilus* NCFM	B	Low G+C Gram positives	
23002679	Lac.gas	*Lactobacillus gasseri*	B	Low G+C Gram positives	97,2% identity with Lac.joh
42519642	Lac.joh	*Lactobacillus johnsonii* NCC 533	B	Low G+C Gram positives	
28379962	Lac.pla	*Lactobacillus plantarum* WCFS1	B	Low G+C Gram positives	
15674137	Lac.la1	*Lactococcus lactis* subsp. lactis	B	Low G+C Gram positives	
27527171	Leu.mes	*Leuconostoc mesenteroides* subsp. cremoris	B	Low G+C Gram positives	
16800743	Lis.inn	*Listeria innocua*	B	Low G+C Gram positives	99,4% identity with Lis.mon
45549371	Lis.iva	*Listeria ivanovii*	B	Low G+C Gram positives	98,6% identity with Lis.mon
16803674	Lis.mon	*Listeria monocytogenes* EGD-e	B	Low G+C Gram positives	
45549373	Lis.see	*Listeria seeligeri*	B	Low G+C Gram positives	99,2% identity with Lis.mon
45549375	Lis.wel	*Listeria welshimeri*	B	Low G+C Gram positives	98,2% identity with Lis.mon
52308297	Man.suc	*Mannheimia succiniciproducens* MBEL55E	B	γ-Proteobacteria	
21435953	Mas.bal	*Mastigamoeba balamuthi*	E	Pelobionts	
23037201	Oen.oen	*Oenococcus oeni* MCW	B	Low G+C Gram positives	
15603318	Pas.mul	*Pasteurella multocida*	B	γ-Proteobacteria	
54308296	Pho.pro	*Photobacterium profundum* SS9	B	γ-Proteobacteria	
37526388	Pho.lum	*Photorhabdus luminescens* subsp. laumondii TTO1	B	γ-Proteobacteria	
33578055	Pir.sp	*Piromyces* sp. E2	E	Fungi	
40644910	Pol.sp.	*Polytomella* sp. Pringsheim 198.80	E	Green algae	
16760134	Sal.ent	*Salmonella enterica* subsp. enterica serovar Typhi	B	γ-Proteobacteria	98,0% identity with Esc.col
16765093	Sal.typ	*Salmonella typhimurium* LT2	B	γ-Proteobacteria	98,1% identity with Esc.col
24373696	She.one	*Shewanella oneidensis* MR-1	B	γ-Proteobacteria	
24112636	Shi.fle	*Shigella flexneri* 2a str. 301	B	γ-Proteobacteria	100,0% identity with Esc.col
27983190	Spi.bar	*Spironucleus barkhanus*	E	Diplomonads	
15923138	Sta.aur	*Staphylococcus aureus* subsp. aureus Mu50	B	Low G+C Gram positives	
27467424	Sta.epi	*Staphylococcus epidermidis* ATCC 12228	B	Low G+C Gram positives	
22536238	Str.aga	*Streptococcus agalactiae* 2603V/R	B	Low G+C Gram positives	95,0% identity with Str.pyo
34787345	Str.bov	*Streptococcus bovis*	B	Low G+C Gram positives	
24378663	Str.mut	*Streptococcus mutans* UA159	B	Low G+C Gram positives	
15901847	Str.pne	*Streptococcus pneumoniae* TIGR4	B	Low G+C Gram positives	
21909572	Str.pyo	*Streptococcus pyogenes* MGAS315	B	Low G+C Gram positives	
50590075	Str.sui	*Streptococcus suis* 89/1591	B	Low G+C Gram positives	

22297771	The.elo	*Thermosynechococcus elongatus* BP-1	B	Cyanobacteria	
58578286	unc.bac	*uncultured bacterium*	B		
15642035	Vib.cho	*Vibrio cholerae* O1 biovar eltor str. N16961	B	γ-Proteobacteria	
59711525	Vib.fis	*Vibrio fischeri* ES114	B	γ-Proteobacteria	
28898895	Vib.par	*Vibrio parahaemolyticus* RIMD 2210633	B	γ-Proteobacteria	95,1% identity with Vib.cho
37679359	Vib.vul	*Vibrio vulnificus* YJ016	B	γ-Proteobacteria	96,9% identity with Vib.cho
16122410	Yer.pes	*Yersinia pestis*	B	γ-Proteobacteria	
