# Supplementary material for: Evolution of four gene families with patchy phylogenetic distributions: influx of genes into protist genomes
Source: BMC Evol Biol. 2006 Mar 21;6:27. doi: 10.1186/1471-2148-6-27 (PMC1484493; doi:10.1186/1471-2148-6-27)
Supplement: Additional File 2 — Information about the datasets and parameters of the phylogenetic analyses. [file 1471-2148-6-27-S2.pdf]

Additional File 2 – Andersson *et al.*

Summary of the datasets used in the phylogenetic analyses.

	priS	*fprA*	*nagB*-short	*nagB-long*	*adhE*
Total number of taxa in dataset	76	125	164	14	68
Number of excluded redundant sequences (>95% identical)	13	15	27	0	18
Number of truncated sequences (<67% of alignment length)	1	1	2	1	0
Number of taxa included in the phylogenetic analysis	62	109	135	13	50
Number of unambiguously aligned aa positions	417	269	229	560	796
Preferred substitution model (ML)	WAG+I+G	WAG+G	WAG+G	RtREV+G	WAG+I+G
P_{inv} (ML)	0.085	0	0	0	0.092
α (ML)	1.319	1.951	1.227	0.871	1.386
Preferred ASRV model (grouped aa)	I+G	I+G	I+G	G	I+G
p_t (grouped aa)	0.46	**0.00**	**0.02**	0.60	**0.00**
P_{sim} (grouped aa)	0.15	**0.00**	**0.00**	0.57	**0.00**
$P_{chi\text{-}square}$ (grouped aa)	1.00	0.15	1.00	1.00	0.89
P_{sim} (original dataset)	**0.00**	**0.00**	**0.00**	**0.00**	**0.00**
$P_{chi\text{-}square}$ (original dataset)	1.00	**0.00**	**0.00**	1.00	1.00

p_t: Values for the MCMC posterior predictive simulations [1]

P_{sim}: P values for composition homogeneity test using simulations to get the null distribution [1]

$P_{chi\text{-}square}$: P values for composition homogeneity test using the χ^2 curve to get the null distribution [1]

References

1. Foster PG: **Modeling compositional heterogeneity.** *Syst Biol* 2004, **53:**485-495.
