# Supplementary material for: Evolution of four gene families with patchy phylogenetic distributions: influx of genes into protist genomes
Source: BMC Evol Biol. 2006 Mar 21;6:27. doi: 10.1186/1471-2148-6-27 (PMC1484493; doi:10.1186/1471-2148-6-27)
Supplement: Additional File 3 — Figures showing the split support for the two runs in the grouped aa analyses plotted one against the other as indicators of convergence. [file 1471-2148-6-27-S3.pdf]

Additional File 3 - Andersson *et al.*

Split support values for the duplicate runs plotted against each other as an indicator of convergence

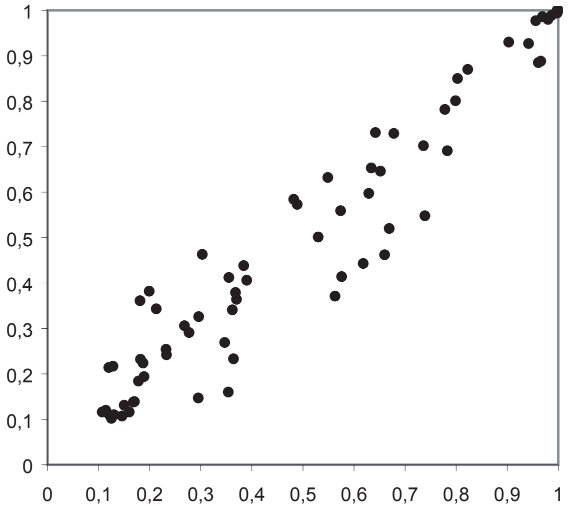

Hybrid-cluster protein (*priS*)
62 taxa, 417 positions

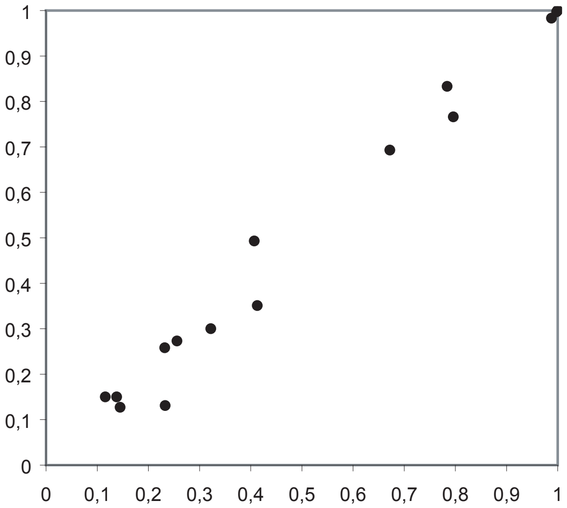

Glucosamine-6-phosphate isomerase (*nagB*)
13 taxa, 560 positions

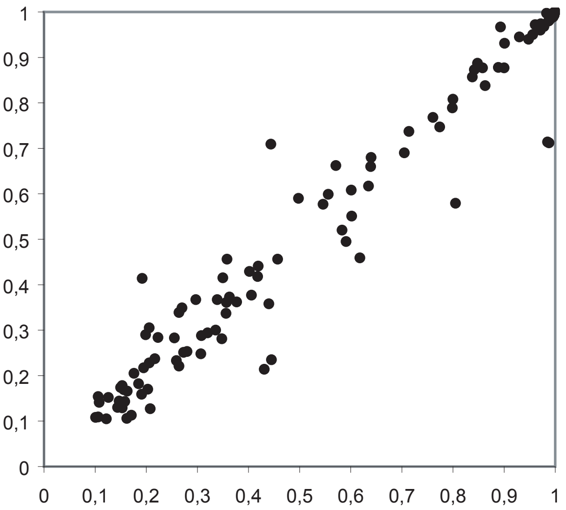

A-type flavoprotein (*fprA*)
109 taxa, 269 positions

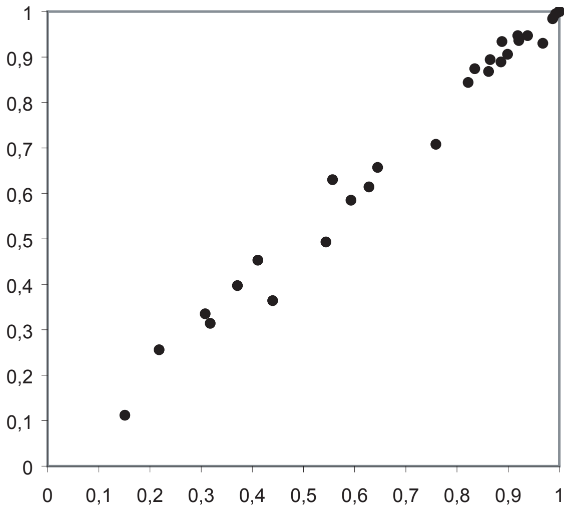

Alcohol dehydrogenase E (*adhE*)
50 taxa, 796 positions

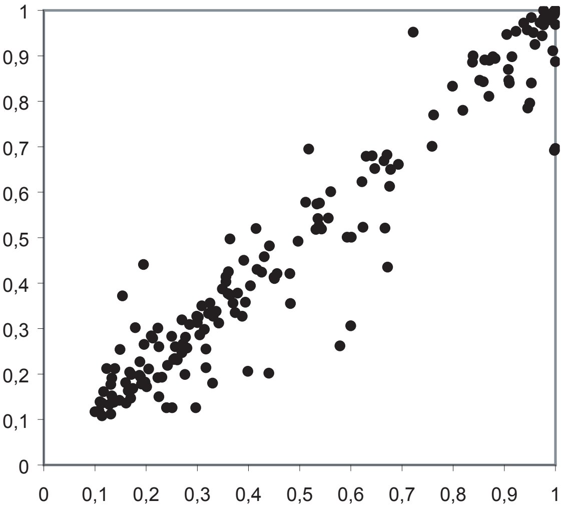

Glucosamine-6-phosphate isomerase (*nagB*)
135 taxa, 229 positions
