# Supplementary material for: Evolution of four gene families with patchy phylogenetic distributions: influx of genes into protist genomes
Source: BMC Evol Biol. 2006 Mar 21;6:27. doi: 10.1186/1471-2148-6-27 (PMC1484493; doi:10.1186/1471-2148-6-27)
Supplement: Additional File 4 — Figure showing the structural organization of the T. vaginalis A-type flavoproteins together with additional discussion. [file 1471-2148-6-27-S4.pdf]

Additional file 4 – Andersson *et al.*

Structural organization of *Trichomonas vaginalis* proteins encoded by the *fprA* genes.

Three *Trichomonas* sequences, a *Clostridium perfringens* sequence and a *Azoarcus* sp. EbN1 sequence share an organization made of two domains corresponding to four entries in the CDD database as shown in the figure. (A) The top diagram is the graphical representation of the RPS-Blast result at the NCBI Blast server (for *T. vaginalis* homolog 2) indicating the position of the four CDD entries on the *Trichomonas* protein. (B) The four CDD entries' name and accession numbers are shown. (C) The N-terminal domain corresponds to A-type flavoprotein and the C-terminus corresponds to a FAD-dependent pyridine nucleotide-disulphide oxidoreductase:Rubredoxin-type or NADH dehydrogenase, similar to nitrite reductase. The numbers indicate the positions of the *T. vaginalis* homolog 2 segments used for independent BlastP analyses. (D) Tables listing the BlastP hits for the top two prokaryotic and top two eukaryotic hits for each domain.

The separation of the *Trichomans* and *Clostridium* A-type flavoproteins proteins from the *Azoarcus* homolog in the phylogenetic analyses (Figure 3) strongly suggests that these domains have fused at least twice independently. However, several scenarios could explain the presence of fused proteins in the *Clostridium* and *Trichomonas* lineages; (i) the two domains could have been transferred independently into the *Trichomonas* lineage and then fused, (ii) the domains could have been fused in a clostridial genome and then transferred into *Trichomonas*, or (iii) the two domains could have been fused earlier and subsequently been lost several times independently in the lineages leading to *Chlorobium*, *Clostridium, Entamoeba*, and *Mastigamoeba*. Unfortunately, the poor resolution in this region of the trees of the phylogenetic analyses of the A-type flavoprotein (see Figure 3 and main text) and the limited knowledge of the likelihoods of gene fusion, gene split and gene transfer events in the evolution of these gene families do not allow differentiating between these scenarios. In any case, the two domains represent candidate LGT events from prokaryotic donors to the parabasalid lineage.

Additional File 4 - Andersson *et al.*

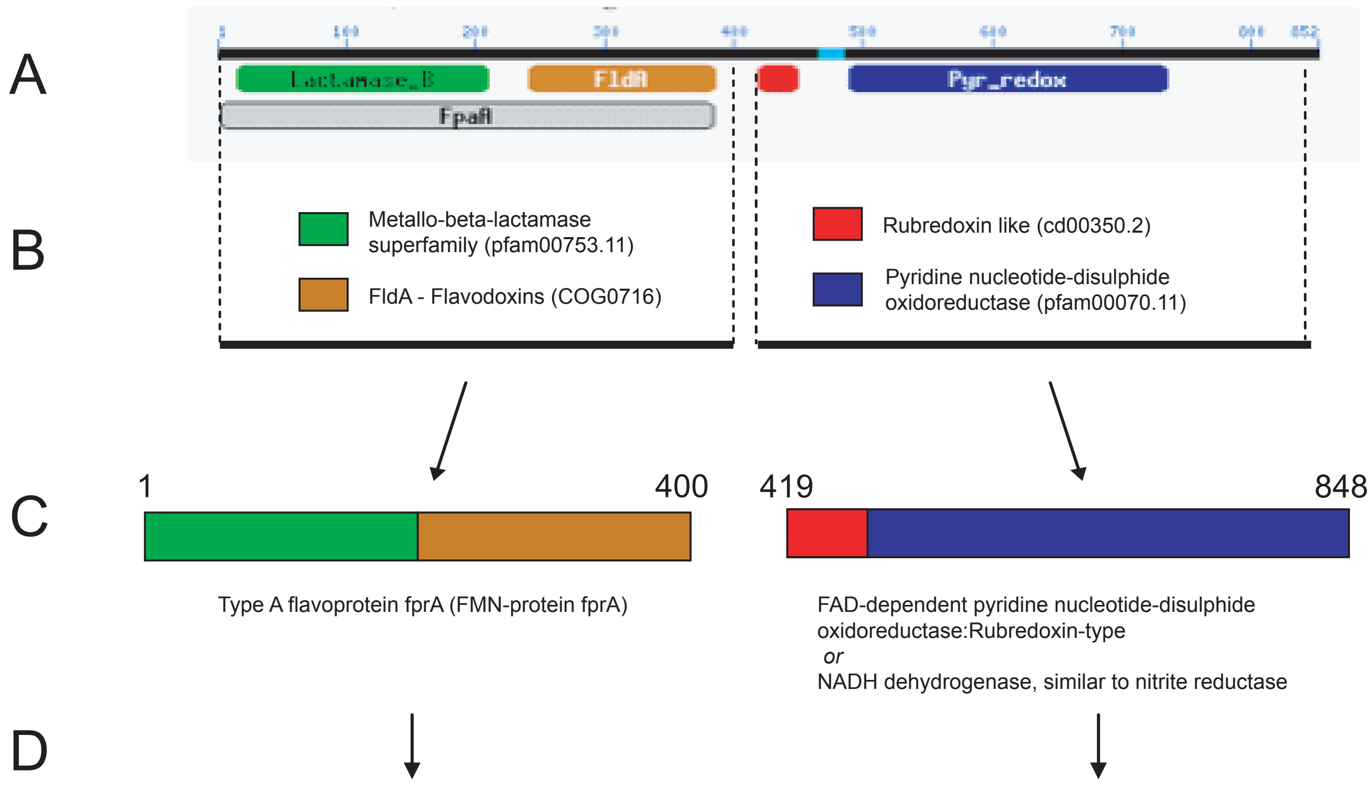

Top two prokaryotic and eukaryotic hits

Taxa	Length/aa	Accession	Score	E-value
Clostridium perfringens	878	BAB80720.1	345	1e-93
Chlorobium phaeobacteroides	425	ZP_00662309.1	286	7e-76
Entamoeba histolytica	406	XP_656946.1	293	9e-78
Giardia lamblia	401	AAM94654.1	119	2e-25

Top two prokaryotic and eukaryotic hits

Taxa	Length/aa	Accession	Score	E-value
Clostridium tetani E88	433	AAO36981.1	302	1e-80
Clostridium perfringens	878	BAB80720.1	298	3e-79
Entamoeba histolytica	452	AAL59604.1	150	8e-35
Aspergillus fumigatus	1102	AL90616.1	132	3e-29

See Figure 3 for phylogeny
