# Supplementary material for: Evolution of four gene families with patchy phylogenetic distributions: influx of genes into protist genomes
Source: BMC Evol Biol. 2006 Mar 21;6:27. doi: 10.1186/1471-2148-6-27 (PMC1484493; doi:10.1186/1471-2148-6-27)

Additional file 5 – Andersson *et al.*

ML analysis of the *nagB* data set with long branch sequences excluded as described in the legend to Figure 4. Same methods and labelling as in Figure 4. Keys to the species names are found in Additional file 1. Numbers at nodes indicate fraction of 500 bootstrap replicates.

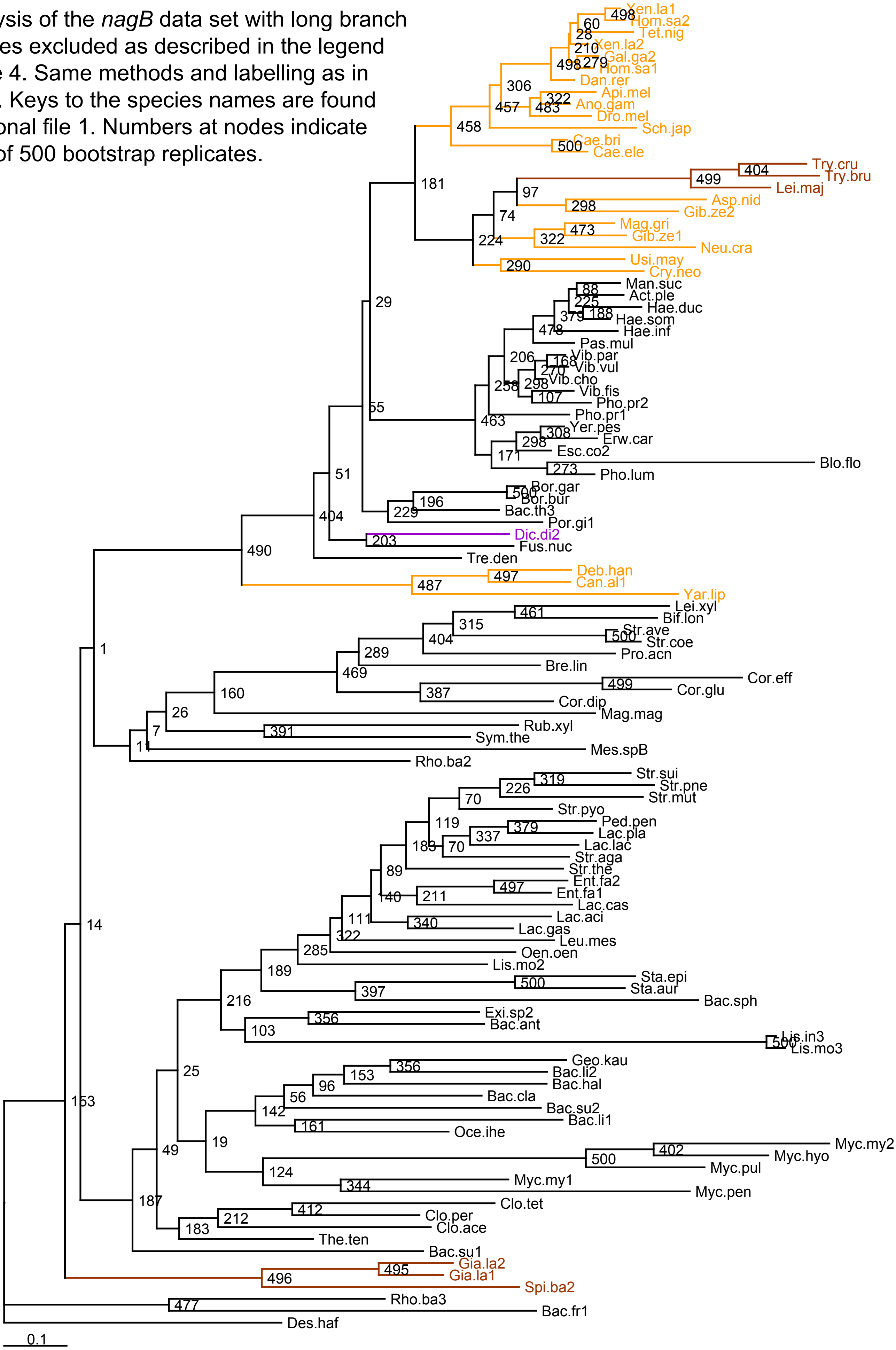

Supplement: Additional File 5 — Figure showing a phylogenetic analysis of the short version of glucosamine-6-phosphate isomerase with the long version and some prokaryotic long branches excluded. [file 1471-2148-6-27-S5.pdf]
